# Phenylboronic Acid-Modified Polyethyleneimine: A Glycan-Targeting Anti-Biofilm Polymer for Inhibiting Bacterial Adhesion to Mucin and Enhancing Antibiotic Efficacy

**DOI:** 10.1021/acsami.4c20874

**Published:** 2025-03-18

**Authors:** Lorcan J. P. Rooney, Andrew Marshall, Michael M. Tunney, Seyed R. Tabaei

**Affiliations:** † School of Chemistry and Chemical Engineering, Queen’s University Belfast, David Keir Building, Stranmillis Road, Belfast BT9 5AG, U.K.; ‡ School of Pharmacy, Queen’s University Belfast, Medical Biology Centre, Lisburn Road, Belfast BT9 7BL, U.K.

**Keywords:** antibiofilm materials, boronic acid-functionalized polymers, glycan-binding nanomaterials, nanoarchitectonics, mucin interaction, Pseudomonas aeruginosa biofilms, synergistic antibiotic activity, polyethyleneimine (PEI)

## Abstract

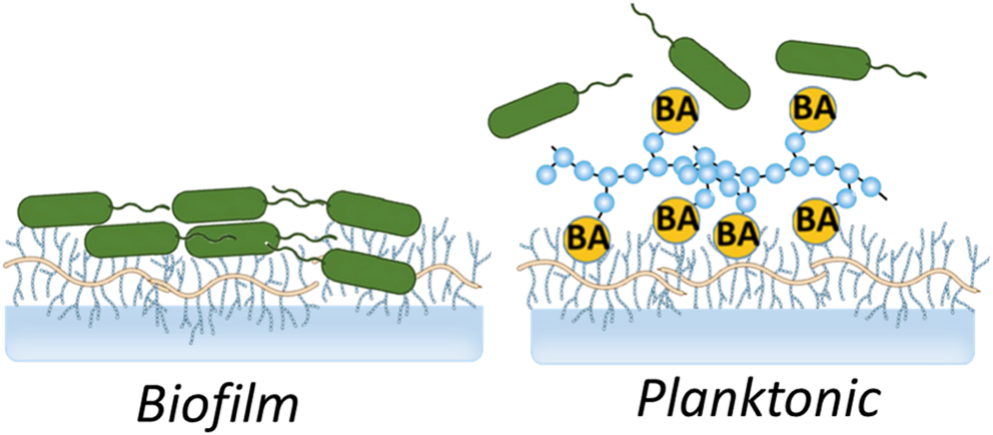

Bacterial biofilms present significant therapeutic challenges due to their resistance to conventional antimicrobial treatment. Mucins typically serve as a protective barrier against pathogens, yet certain bacteria, such as *Pseudomonas aeruginosa* (*P. aeruginosa*), can exploit these glycoproteins as attachment sites for biofilm formation. This study introduces boronic acid-functionalized polyethyleneimine (PEI-BA) as a promising antibiofilm agent that effectively blocks bacterial adhesion to mucin-rich surfaces. Through the multivalent presentation of boronic acid groups, PEI-BA reversibly forms boronate ester bonds with mucin glycans, creating a protective barrier. Our findings show that PEI-BA prevents bacterial attachment through a nonbactericidal mechanism, potentially reducing the risk of resistance development. Notably, PEI-BA synergizes with a conventional antibiotic, tobramycin, significantly enhancing biofilm inhibition compared to either treatment alone. Systematic evaluation of PEI-BA formulations identified optimal functionalization levels, balancing glycan-binding capability with solubility. From a biomaterials design perspective, we demonstrate how rational polymer modification can transform a potent but cytotoxic antimicrobial agent (i.e., PEI) into a safe and effective antibiofilm material, opening further possibilities for managing biofilm-associated infections in clinical settings. This work establishes boronic acid-based nanomaterials as promising candidates for biofilm prevention and antibiotic enhancement, particularly in conditions like cystic fibrosis, where mucin-bacterial interactions contribute to disease progression.

## Introduction

The assembly of microorganisms and their subsequent development into biofilms pose a significant therapeutic challenge, largely due to enhanced resistance to conventional antimicrobial treatment. At the molecular level, biofilms form a protective barrier through their extracellular polymeric substance (EPS) matrix, which shields bacteria from both antibiotics and immune defenses.^1^ This barrier not only limits antibiotic penetration but also promotes bacterial resilience, increasing antibiotic resistance by a factor of 10–1000.^2^ These characteristics contribute to the persistent, chronic, and recurrent nature of biofilm-associated infections, making them particularly difficult to treat.^3^

The initial interfacial interaction between bacterial surfaces and mucins is of particular interest in understanding pathogen colonization.^4^ Mucins, a family of heavily glycosylated proteins, are the primary components of the mucus layer that protects epithelial cells in various human organs.^5^ They form a highly cross-linked and entangled polymeric network that functions as a selective barrier, with their main role being to trap and facilitate the clearance of pathogens, thereby preventing unwanted interactions with the underlying cells. Under normal physiological conditions, pathogens bound to mucins are efficiently removed through the regular turnover of the mucus layer.^6^ Additionally, the highly hydrated mucin network functions as a bacterial repellent, and this property has been successfully exploited to create pathogen-resistant barriers in various applications.^7^ However, in conditions like cystic fibrosis (CF), characterized by dehydrated mucus and airway obstruction, pathogens such as *Pseudomonas aeruginosa* (*P. aeruginosa*) can exploit mucins as stable attachment sites.^8^ This exploitation increases bacterial residence time, enhancing the likelihood of biofilm formation.

The interfacial interactions between bacterial cells and mucin molecules play a crucial role in pathogenesis, yet they remain incompletely understood. Studies have shown that this binding process is mediated, at least in part, by specific carbohydrate-binding proteins, known as lectins, located on the bacterial surface.^9,10^ These lectins recognize and bind to glycan structures on mucin molecules, facilitating the attachment of bacteria to mucosal surfaces. This interaction between bacterial adhesins and mucin glycoproteins represents a critical initial step in pathogenesis and is an important therapeutic target for preventing biofilm-associated infections.

One strategy to prevent bacterial attachment is to target bacterial lectins using small molecule inhibitors or glycomimetic antagonists, compounds designed to mimic host glycans and competitively inhibit lectin binding.^11−15^ However, this approach faces significant challenges due to the complexity of glycan-lectin interactions, which involve specific binding preferences and structural recognition that vary widely among bacterial species. Additionally, mucin glycoproteins have a highly heterogeneous range of glycosylation patterns, making it difficult to develop a single glycomimetic antagonist capable of effectively blocking all relevant lectin-glycan interactions across different mucin types and bacterial strains.

While all lectins require glycan residues to recognize and bind to their macromolecular ligands, the specific glycoproteins or glycolipids involved in lectin binding are often unknown due to the remarkable diversity of glycan structures. Glycan research is notably more complex and less developed compared to genome and protein research,^16^ and the specific glycans that interact with bacterial lectins have yet to be fully identified.^17^ Systematic studies on lectin discovery and their therapeutic applications are still limited, and more research is needed to map glycan-lectin interactions and understand their roles in disease.

Furthermore, the large-scale production of glycomimetic drugs remains challenging and costly, presenting additional obstacles to this approach.^18^ Overall, although targeting bacterial lectins with glycomimetic antagonists holds potential, substantial work is needed to address these limitations and advance this strategy for effective disease prevention.^19^

An alternative strategy to prevent bacterial attachment is targeting mucins directly, rather than lectins. *In vitro* studies have shown that blocking sialylated mucins with antibodies can inhibit *P. aeruginosa* binding and reduce infection, particularly in CF airways.^20^ This approach aims to disrupt bacterial adhesion by blocking the host glycoproteins that bacteria exploit for attachment. However, multiple lectins are typically involved in bacterial binding, so a single antibody may not suffice, potentially requiring a combination for full inhibition. Additionally, due to the low immunogenicity of mammalian glycan structures, producing highly specific anti-glycan antibodies can be challenging.^21^

To address these limitations, this work aims to utilize a phenylboronic acid as a versatile glycan-binding moiety.^22^ Boronic acid (BA) derivatives are widely used recognition elements for binding *cis*-diol-containing biomolecules, such as glycans with multiple hydroxyl groups.^23^ BAs form reversible five- or six-membered cyclic esters with 1,2- or 1,3-*cis*-diol-containing compounds. While individual BAs show moderate affinity for *cis*-diols (ranging from 10^–1^ to 10^–3^ M),^24^ their functionality becomes significantly enhanced when incorporated into multivalent scaffolds. As such, a variety of BA-functionalized nanomaterials have been developed for applications in the recognition, extraction, and separation of glycans.^25−29^

**Figure 1:**
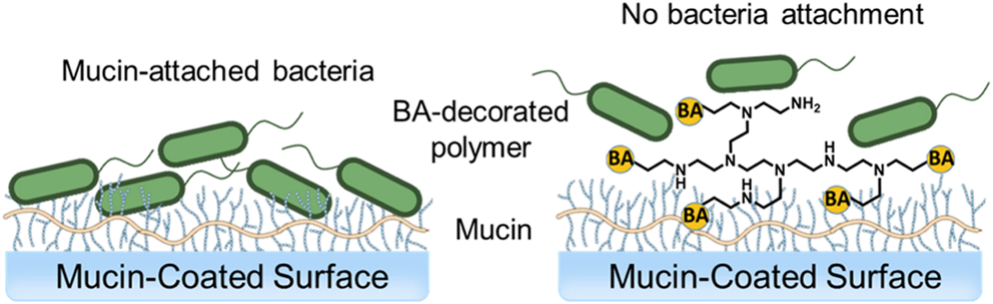
Schematic representation of the proposed approach utilizing a mucin-binding polymer to prevent bacterial adhesion to surfaces. *P. aeruginosa* can adhere to complex carbohydrate structures on mucins, which is a critical initial step in the formation of persistent biofilms. Boronic acids (BAs) can form reversible complexes with polyols, including sugars and heavily glycosylated proteins like mucins. By decorating polymers with BA, these materials can effectively compete with bacteria for binding sites on mucin, thereby reducing bacterial adhesion and colonization on mucosal surfaces.

We hypothesize that the ability of BAs to form dynamic covalent interactions with diols in glycan structures can be leveraged to design a nanomaterial capable of competitively binding to mucins. This binding is expected to form a protective barrier that inhibits bacterial access to natural binding sites, thereby disrupting bacterial colonization and biofilm development (Figure 1).

Nanoarchitectonics,^30,31^ the engineering of functional materials at the molecular and nanoscale level, offers innovative solutions to combat biofilms.^32^ By precisely designing surfaces,^33^ creating targeted drug delivery systems,^34^ and developing biofilm disruption strategies,^35^ researchers are leveraging nanotechnology to overcome the challenges posed by resilient microbial communities. In particular, the development of polymer-based strategies for biofilm disruption has emerged as a promising approach, encompassing various mechanisms including targeting physical (e.g., the electrostatic interaction with the negatively charged bacterial surfaces),^36^ mechanical (e.g., viscoelastic properties of the biofilm),^37^ and chemical aspects (e.g., membrane-lysis mechanism)^38^ of biofilm formation. Building on these principles, herein, we report the development of a biocompatible boronic acid-functionalized polyethyleneimine polymer (PEI-BA) and demonstrate its robust antibiofilm activity against *P. aeruginosa* at glycan-rich mucin interfaces. Furthermore, we highlight a notable synergistic effect when the PEI-BA nanomaterial is combined with the conventional antibiotic tobramycin, underscoring its potential as a complementary strategy for managing bacterial infections.

## Results and Discussion

To harness the broad glycan-binding capacity of BAs, we incorporated BAs onto a branched polyethyleneimine (PEI) polymer, creating a multivalent scaffold. We employed reductive amination to conjugate 3-fluoro-4-formylphenylboronic acid (3-BA) onto 1.8 kDa branched polyethyleneimine. This synthetic approach effectively converts aldehydes to amines via imine intermediates, providing a reliable method for boronic acid functionalization of the polymer. The branched architecture of PEI is an ideal multivalent scaffold for boronic acid functionalization, offering numerous accessible amine sites for conjugation. This structure not only increases the number of boronic acid moieties but also maintains conformational flexibility. PEI has been widely utilized in the generation of polymer-mediated nanomaterials.^39−42^

**Scheme 1:**
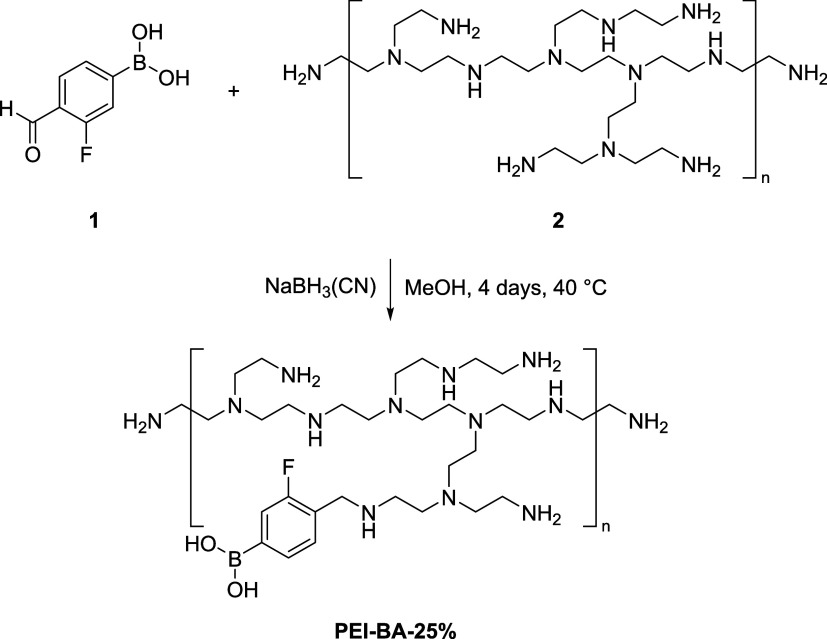
Synthesis of Polyethyleneimine-boronic Acid (PEI-BA) Nanomaterials The reaction involves the conjugation of 3-fluoro-4-formylphenylboronic acid (3-BA) (1) with 1.8 kDa branched polyethyleneimine (PEI) (2) via reductive amination using sodium cyanoborohydride (NaBH3(CN)) as the reducing agent in methanol (MeOH) over 4 days at 40 °C. Nanomaterials with varying molar ratios of boronic acid/PEI monomer (0.4:1, 1:1 & 2:1) were synthesized

To systematically investigate how the degree of boronic acid functionalization affects glycan binding, we synthesized three distinct PEI-BA nanomaterials with increasing BA:monomer molar ratios (0.4:1, 1:1, and 2:1) as shown in Scheme 1. These materials are designated as PEI-BA-10%, PEI-BA-25%, and PEI-BA-50%, respectively, based on the assumption that conjugation occurs predominantly at the primary amine sites of branched PEI monomers. The rationale for exploring different functionalization levels is to determine whether increased boronic acid coverage would enhance interactions with mucin’s carbohydrate structures, potentially leading to improved binding efficiency. This multivalent design strategy is expected to effectively transform relatively weak individual boronic acid-diol interactions into strong, stable complexes through the collective contribution of multiple binding events, where the spatial distribution and density of boronic acid groups can significantly impact overall binding efficiency.

**Figure 2:**
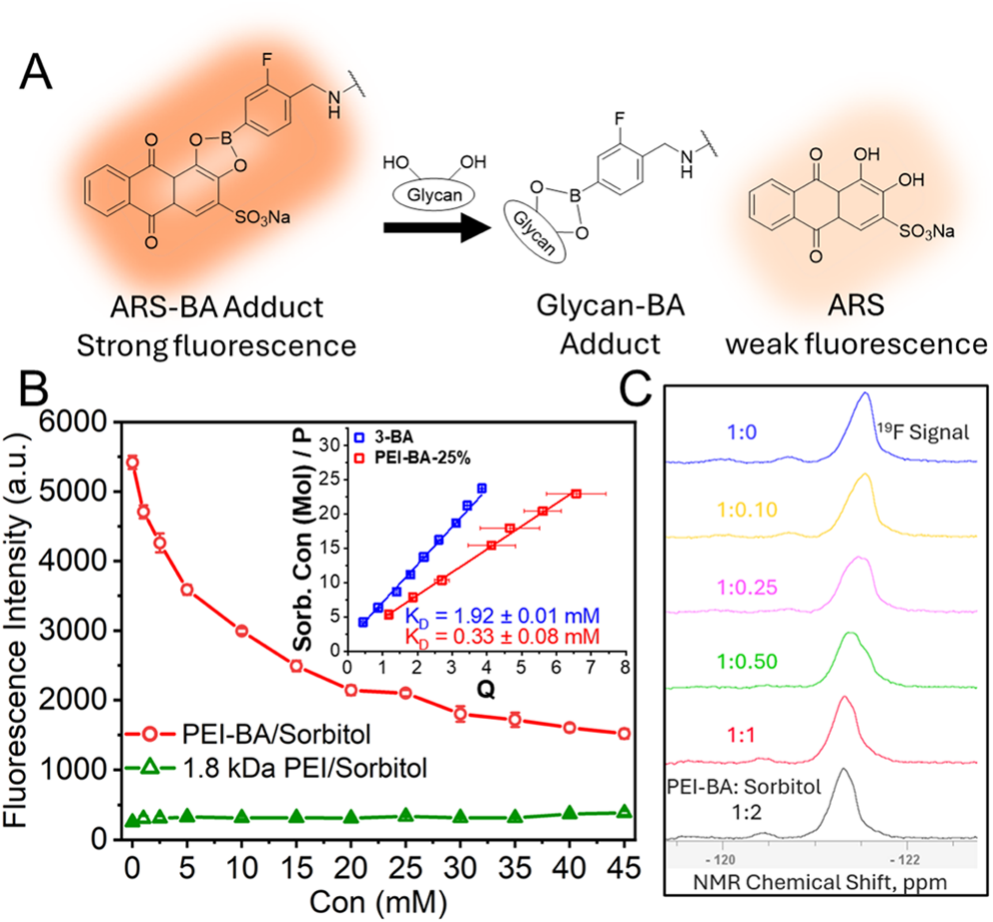
Evaluation of glycan binding to PEI-BA nanomaterials. (A) Schematic representation of the alizarin red S (ARS) assay. The ARS-BA adduct shows strong fluorescence. Upon interaction with glycans, the boronic acid binds to 1,2- or 1,3-*cis*-diols present in the glycan structure, displacing ARS and forming a glycan-BA adduct. This reaction leads to the release of free ARS, which displays significantly weaker fluorescence. (B) Competitive binding of PEI-BA with sorbitol compared to 1.8 kDa PEI (control), monitored by ARS fluorescence intensity as a function of sorbitol concentration. PEI-BA exhibits a significant decrease in fluorescence, indicating strong sorbitol binding, whereas 1.8 kDa PEI shows negligible interaction. The inset shows analysis for the determination of binding affinities of 3-BA and PEI-BA-25%, revealing dissociation constants (*K*_D_) of 1.92 ± 0.01 and 0.33 ± 0.08 mM, respectively (see Supporting Information for detailed analysis). (C) The ^19^F NMR spectra shows a disappearance of the ^19^F peak of the 3-BA moiety in the absence of sorbitol, and an appearance of a new, downfield-shifted ^19^F peak as the ratio of sorbitol monomer increases from 0:1 to 1:1. The dashed line represents the initial position of the ^19^F peak in the absence of sorbitol.

To validate the glycan-binding capability of the synthesized PEI-BA nanomaterials, we employed an alizarin red S (ARS) competitive binding assay (Figure 2A).^43,44^

To characterize our system’s binding behavior, we employed sorbitol as a model compound (Figure 2B). Sorbitol shows relatively strong affinity for boronic acid derivatives and serves as a suitable model compound in boronic acid-based glycan recognition studies.^24^

Initial binding studies using an ARS displacement assay demonstrated that titration of sorbitol into PEI-BA/ARS complexes resulted in concentration-dependent fluorescence decline, achieving approximately 70% reduction in signal intensity. Similarly, another model monosaccharide (i.e., fructose) showed a concentration-dependent fluorescence decline (Figure S7). The specificity of the interaction was confirmed by the absence of fluorescence changes when using unmodified PEI (green curve), which lacks boronic acid groups. To validate our multivalent design strategy, we used the ARS competitive binding assay to measure and compare the binding affinity for sorbitol between PEI-BA and free 3-BA. The results (Figure 2B, inset) demonstrated that PEI-BA exhibited a more than 6-fold greater affinity for sorbitol (*K*_D_ = 0.33 mM) compared to that of free 3-BA (*K*_D_ = 1.92 mM). This comparison highlights the enhanced binding efficiency achieved through our multivalent design.

To further validate these findings and directly probe the molecular basis of glycan recognition, we employed ^19^F NMR spectroscopy, a technique previously established for monitoring glycan binding to fluorinated boronic acids.^45,46^ Using a titration assay, we monitored changes in the appearance and chemical shift of the peak of the fluorine atom in the 3-BA moiety while gradually increasing the sorbitol:PEI-BA ratio from 0:1 to 1:1. As shown in Figure 2C, the initial ^19^F signal at approximately −121.5 ppm begins to decrease in intensity, while a new, more downfield ^19^F signal at approximately −121.3 ppm begins to appear with increasing sorbitol concentration, reaching its maximum displacement at a 1:1 ratio. The disappearance of the original ^19^F signal and the shifted emergence of a new ^19^F signal upon introduction of sorbitol reflects changes in the local electronic environment of the boronic acid group upon interaction with sorbitol’s *cis*-diols, providing direct evidence that the boronic acid moieties are responsible for glycan recognition and binding.

Furthermore, the glycan-binding efficiency of PEI-BA nanomaterials showed a direct correlation with their boronic acid content. Nanomaterials with higher BA:PEI monomer ratios demonstrated greater maximum fluorescence intensities upon ARS addition (Figure S4), confirming that increased boronic acid functionalization enhances multivalent interactions with glycan targets. Collectively, these results provide comprehensive evidence for both the successful functionalization of PEI with boronic acid groups and their ability to specifically recognize and bind *cis*-diol-containing molecules, a key structural feature of glycans.

**Figure 3:**
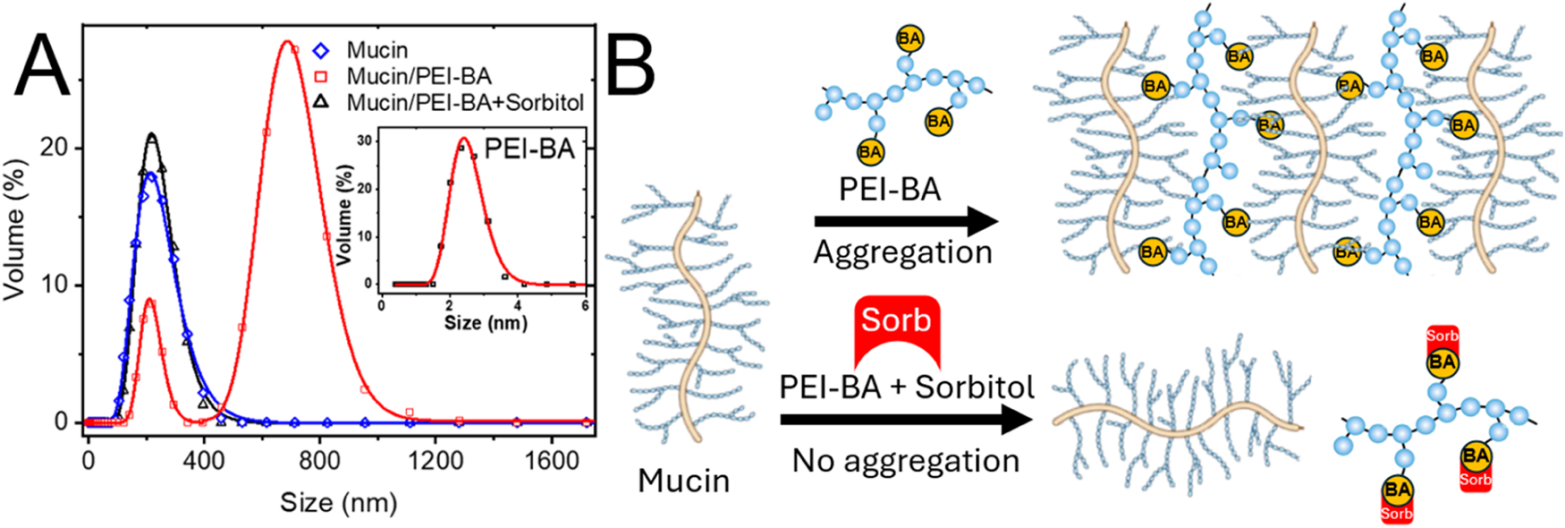
Mucin aggregation analysis using dynamic light scattering (DLS). (A) The DLS data show the size distribution of mucin (blue), mucin mixed with PEI-BA (red), and mucin mixed with PEI-BA and sorbitol (black). The addition of PEI-BA to mucin resulted in a significant increase in particle size from ∼200 to ∼800 nm, indicating mucin aggregation due to boronic acid-glycan interactions. In contrast, when sorbitol was included in the mixture, the particle size distribution resembled that of mucin alone, suggesting sorbitol competes for boronic acid binding sites, preventing mucin aggregation. The inset shows the DLS data for PEI-BA-25% alone, with an average size of 2.3 nm, confirming its nanoscale dimensions. (B) Schematic illustration of PEI-BA-mediated mucin aggregation and its modulation by sorbitol. The competitive binding mechanism illustrates the specificity of the boronic acid-glycan interactions.

Next, dynamic light scattering (DLS) was employed to investigate the ability of PEI-BA to bind and cross-link glycosylated mucin proteins (Figure 3A). Initial characterization revealed that PEI-BA exists as small nanoscale particles (<5 nm) (Figure 3A, inset), while mucin alone shows a characteristic size distribution centered at approximately 200 nm. Upon the addition of PEI-BA to mucin solutions, significant aggregation occurred, evidenced by a 4-fold increase in particle size to approximately 800 nm (Figure 3A). This aggregation behavior showed both concentration- and time-dependent characteristics, with larger aggregates forming at higher PEI-BA-25% concentrations and over time (Figures S11 and S12). The observed aggregation can be attributed to multivalent cross-linking interactions (Figure 3B). In this mechanism, multiple boronic acid moieties on each PEI-BA molecule form boronate ester bonds with glycans on different mucin proteins. The heavily glycosylated nature of mucin enables extensive cross-linking, where individual mucin molecules can simultaneously interact with multiple PEI-BA chains while each PEI-BA polymer bridges several mucin molecules. To confirm the specificity of these boronic acid-glycan interactions, we conducted competitive binding studies using sorbitol. Addition of sorbitol to the mucin/PEI-BA mixture prevented aggregation, maintaining the particle size distribution at approximately 200 nm, characteristic of un-cross-linked mucin. This demonstrates that sorbitol successfully competes for boronic acid binding sites, preventing mucin cross-linking and confirming the boronic acid-dependent nature of the aggregation process.

Having demonstrated PEI-BA’s effective binding to mucin, we next evaluated its potential as an antibiofilm agent. We hypothesized that the interaction of PEI-BA with mucin could create a protective barrier, physically preventing *P. aeruginosa* from accessing mucin glycans that are essential for bacterial adhesion and subsequent biofilm development. To test this hypothesis, we established an *in vitro* model using mucin-coated polystyrene plates to mimic the glycan-rich surface of mucosal tissues. *P. aeruginosa* attachment to such systems has been previously reported, making this a validated model for our studies.^47^

**Figure 4:**
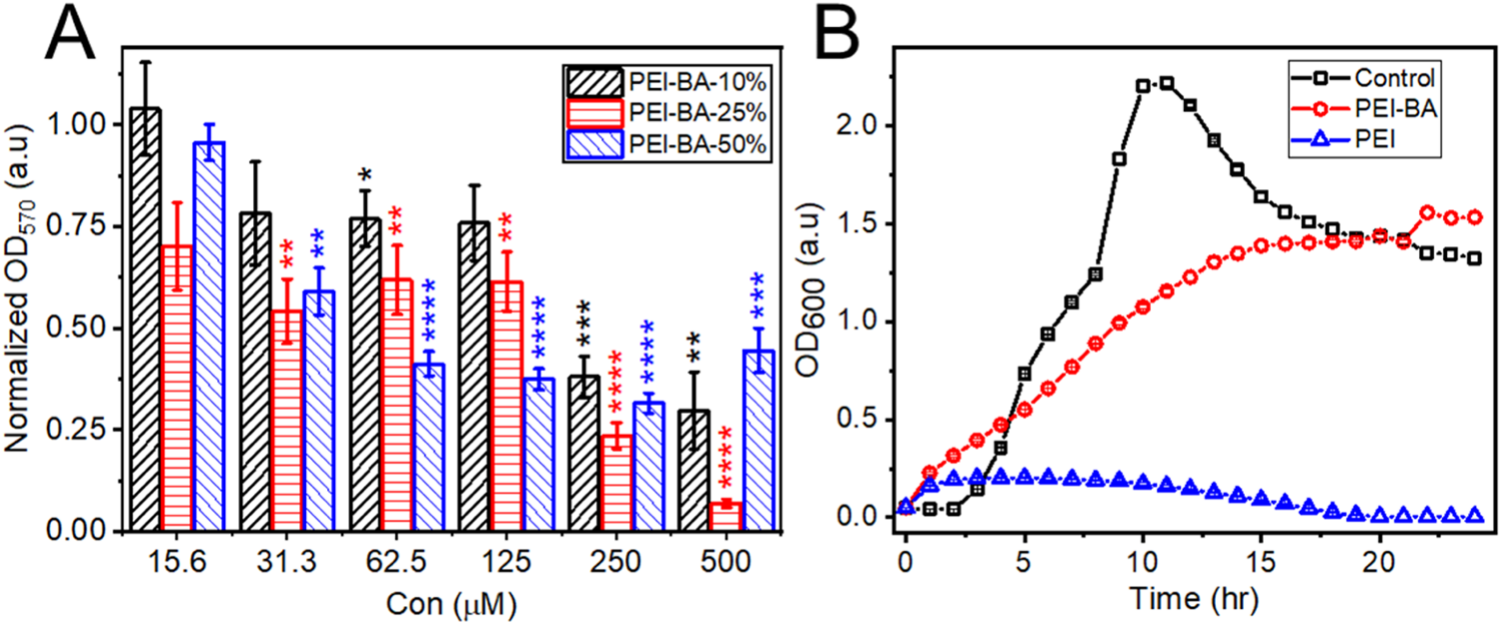
Antibiofilm activity and bacterial growth effects of PEI-BA nanomaterials. (A) Concentration-dependent inhibition of *P. aeruginosa* biofilm formation by PEI-BA with varying degrees of boronic acid modification: PEI-BA-10% (black), PEI-BA-25% (red), and PEI-BA-50% (blue). Biofilms were allowed to form on mucin-coated polystyrene plates. Biofilm formation was quantified by crystal violet staining (OD570) and normalized to untreated controls. The biofilm data are normalized to the untreated control (mucin-coated plates incubated with *P. aeruginosa* only, with no polymer added), which is set to 1.0. (B) Growth kinetics of *P. aeruginosa* in the presence of PEI-BA-25% (0.125 mM, red) compared to unmodified PEI (1 mM, blue) and untreated control (black). The distinct growth patterns demonstrate PEI-BA’s nonbactericidal nature in contrast to the cytotoxic effects of unmodified PEI.

We established mucin coatings through the incubation of polystyrene substrates with mucin solutions, allowing adsorption through hydrophobic interactions between the surface and the mucin protein core, as previously demonstrated.^48^ Bacterial attachment and biofilm formation were then quantitatively assessed by staining the attached bacteria with crystal violet followed by spectrophotometric analysis. All PEI-BA formulations exhibited concentration-dependent inhibition of biofilm formation (Figure 4A), with higher concentrations generally achieving greater than 50% reduction in biofilm formation.

The degree of biofilm inhibition varied among PEI-BA formulations in correlation with their BA content. PEI-BA-10%, with its lower BA modification, consistently showed reduced inhibition compared to PEI-BA-25% and PEI-BA-50% across most concentrations tested. However, this trend deviated at higher concentrations (≥500 μM), where PEI-BA-50% unexpectedly showed decreased efficacy despite its higher BA content. This concentration-dependent behavior reflects a critical balance between BA functionalization and solubility. As BA modification increases, the replacement of positively charged amines with hydrophobic phenylboronic acid groups progressively reduces the polymer’s aqueous solubility at physiological pH. For PEI-BA-50%, this solubility limitation becomes particularly pronounced at higher concentrations, impairing its interaction with the mucin layer and consequently reducing its ability to inhibit biofilm formation. In contrast, at lower concentrations where solubility is maintained, PEI-BA-50%’s higher BA content enables superior biofilm inhibition. To further investigate the solubility challenge of PEI-BA-50%, the particle size of the polymer at both low and high concentrations was measured using DLS and compared to that of PEI-BA-25% (Figure S13). At PEI-BA-25% concentrations of 15.6 μM and 500 μM, the particle size remained consistently below 10 nm, highlighting its improved solubility and stability under these conditions. In contrast, at PEI-BA-50% concentrations of 15.6 and 500 μM, the particle size increased dramatically by over 18-fold from 190 to 3580 nm, respectively. Notably, even at the lower concentration of 15.6 μM, the particle size of PEI-BA-50% was significantly larger than that of PEI-BA-25%, presumably due to the reduced hydrophilicity of the polymer. This substantial increase in particle size led to aggregation, causing the sample to precipitate out of solution. These findings highlight the importance of optimizing BA functionalization: while higher BA content enhances glycan binding and biofilm inhibition, excessive modification can compromise the material’s solubility and thereby its effectiveness.

To further validate the role of boronic acid interactions in biofilm inhibition, we conducted competitive binding studies using sorbitol. The addition of sorbitol (20 mM) alongside PEI-BA-25% led to a significant increase in biofilm formation (Figure S15). Importantly, previous studies have shown that sorbitol itself does not promote bacterial growth; in fact, it may slightly inhibit *P. aeruginosa* growth after 20 h.^49^ Therefore, the increased biofilm formation observed in our studies can be attributed solely to the interaction of sorbitol with PEI-BA rather than any growth-promoting effects.

This observation parallels the aggregation studies (Figure 3), where sorbitol similarly disrupted the interaction of PEI-BA with mucin by competing for boronic acid binding sites. In both cases, sorbitol competitively “caps” the boronic acid sites, preventing interaction between PEI-BA and mucin glycans, thereby allowing *P. aeruginosa* to access the mucin surface and establish biofilms.

The superior performance of higher BA-modified polymers suggests that boronic acid content, rather than the presence of positively charged amines on the polymer backbone, is the primary factor in reducing biofilm formation. This observation is particularly noteworthy because amines are traditionally associated with antibacterial activity through their interaction with negatively charged bacterial cell membranes.^50−52^ Our results instead indicate that the glycan-binding capability of boronic acid moieties plays the dominant role in the antibiofilm activity of PEI-BA. To confirm that the multivalent presentation of BA groups on the polymer backbone is crucial for effective biofilm prevention, we performed control experiments using the small-molecule precursor 3-BA to validate our multivalent design strategy. As expected, free boronic acid groups exhibited no biofilm inhibition at concentrations comparable to the BA groups in PEI-BA (Figure S14).

To distinguish between antiadhesion effects and potential bactericidal activity, we evaluated *P. aeruginosa* growth kinetics in the presence of our materials. Growth curves over a 24-h period (Figure 4B) compared bacterial proliferation in the presence of PEI-BA-25% (red) and PEI (blue) against an untreated control (black). The growth curves revealed distinct effects of PEI and PEI-BA on bacterial viability. Unmodified PEI (blue) almost completely suppressed bacterial growth throughout the 24-h period, demonstrating strong bactericidal activity. This cytotoxicity aligns with PEI’s known mechanism of action, where its high density of positively charged amine groups disrupts bacterial membrane integrity through electrostatic interactions.^53^ In contrast, PEI-BA-25% exhibited markedly different behavior, characteristic of a nonbactericidal material.^54^ While it initially slowed bacterial growth rate, it did not prevent eventual proliferation. Instead of the typical rapid growth and death phases observed in untreated cultures (black), PEI-BA-25%-treated bacteria showed a slower, sustained growth pattern. By the 24-h time point, bacterial populations in PEI-BA-25%-treated samples reached levels comparable to the control, confirming that BA modification transforms PEI from a bactericidal agent to a growth-delaying compound with minimal cytotoxicity. In accordance with previously published reports demonstrating that phenylboronic acid functionalization does not adversely affect the cytocompatibility of low-molecular-weight PEI (PEI_1.8k_),^55^ we specifically chose to work at PEI-BA concentrations below 20 μg/mL. Prior studies have shown that concentrations of 20–100 μg/mL do not significantly reduce the viability of MCF-7 cells,^55^ indicating a robust safety margin. Thus, by operating well within this established safety range, we ensured minimal cytotoxic risk while preserving the material’s antibiofilm efficacy.

While numerous strategies and biomaterials have been developed to combat *P. aeruginosa* infection,^9,56−60^ direct targeting of bacteria can potentially lead to resistance over time. Therefore, preventing biofilm formation by blocking the attachment of planktonic bacteria offers an alternative approach that may reduce the risk of resistance development. *P. aeruginosa* is known to utilize multiple adhesion mechanisms, including the lectins LecA and LecB.^61^ Elucidating the precise molecular pathways of our polymer’s interaction—such as whether it directly interferes with these lectins—represents an important direction for future studies.

**Figure 5:**
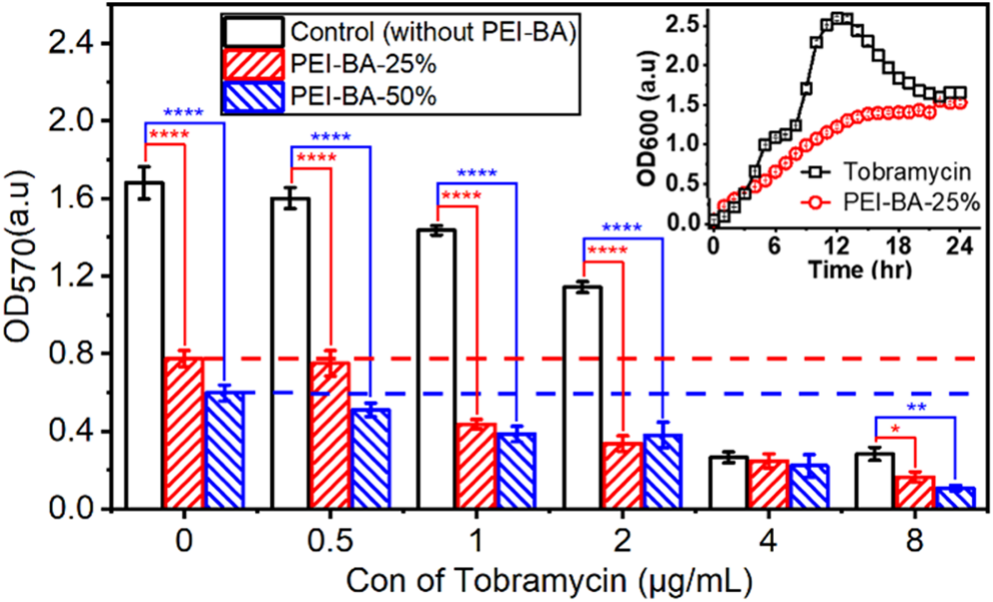
Synergistic inhibition of *P. aeruginosa* biofilm formation by PEI-BA and tobramycin combinations. (A) Antibiofilm activity of PEI-BA variants combined with tobramycin. Biofilm formation was quantified in response to PEI-BA-25% (100 μM, red bars) or PEI-BA-50% (100 μM, blue bars) combined with increasing tobramycin concentrations (0–8 μg/mL). Horizontal dotted lines indicate baseline antibiofilm activity of PEI-BA alone (red: PEI-BA-25%; blue: PEI-BA-50%). The inset presents the growth kinetics of *P. aeruginosa* exposed to PEI-BA-25% at 100 μM and tobramycin at 1 μg/mL, tested individually rather than in combination. The observed growth patterns indicate that neither component exhibits bactericidal activity at these concentrations.

Given the protective environment that biofilms provide against traditional antibiotic treatments, we hypothesized that combining biofilm prevention with antibiotic therapy could enhance overall treatment efficacy. To test this hypothesis, we investigated potential synergistic effects between PEI-BA and tobramycin, an antibiotic commonly prescribed for treatment of *P. aeruginosa* infection (Figure 5).^29,62−64^ Tobramycin, a bactericidal antibiotic, kills *P. aeruginosa* through multiple mechanisms, including disruption of bacterial protein synthesis and cell membrane integrity.^65^ However, its effectiveness can be significantly compromised when bacteria reside within biofilms or at lower antibiotic concentrations.^66^

To investigate the combined effects of PEI-BA and tobramycin on *P. aeruginosa* biofilm formation, biofilm density was measured across increasing concentrations of tobramycin (0–8 μg/mL) in the presence or absence of PEI-BA. Two variants of PEI-BA (PEI-BA-25% and PEI-BA-50%) were included at a fixed concentration of 100 μM to assess dose-dependent effects within the PEI-BA component. The control groups (without PEI-BA) showed that tobramycin alone exhibited a dose-dependent inhibition of biofilm formation, as indicated by the decrease in OD values with increasing antibiotic concentrations (Figure 5). However, substantial biofilm formation remained at lower tobramycin concentrations, indicating that tobramycin alone has limited antibiofilm efficacy at subinhibitory doses. When used alone, PEI-BA-25% and PEI-BA-50% also demonstrated moderate antibiofilm activity, represented by the horizontal red and blue dashed lines, respectively (Figure 5). These baseline activities indicate the independent biofilm inhibition potential of each PEI-BA variant, with PEI-BA-50% showing a slightly higher inhibition compared to PEI-BA-25%. When combined with tobramycin, both PEI-BA-25% and PEI-BA-50% significantly enhanced biofilm inhibition compared to either agent alone, demonstrating a clear synergistic effect. This synergy was particularly pronounced at lower tobramycin concentrations (1–2 μg/mL). At a sub-bactericidal concentration of tobramycin (1 μg/mL), the combination with PEI-BA-25% reduced biofilm formation to approximately 30% of the control level (without PEI-BA). While complete biofilm eradication remains the ultimate goal, achieving a 70% reduction might be clinically significant when combined with other treatments and the host’s natural immune response. Importantly, at these concentrations, neither PEI-BA nor tobramycin alone exhibits bactericidal effects (Figure 5, inset); however, their combination significantly prevents biofilm formation. This synergistic interaction suggests a complementary mechanism where PEI-BA’s antiadhesion properties work together with sub-bactericidal levels of tobramycin to effectively inhibit biofilm establishment. Our data suggests that PEI-BA’s ability to prevent biofilm formation results in *P. aeruginosa* remaining in a more vulnerable, planktonic state, enhancing its susceptibility to the bactericidal effect of tobramycin.

This synergistic interaction is of notable clinical relevance. Biofilms are known to enhance bacterial tolerance to antibiotics, often necessitating higher antibiotic doses to achieve effective treatment outcomes. The observed synergy suggests that combining PEI-BA with tobramycin could achieve substantial biofilm inhibition at lower antibiotic concentrations, potentially reducing the risk of toxicity and delaying the development of antibiotic resistance. Furthermore, the polymer-based nature of PEI-BA makes it amenable to various localized delivery strategies, such as inhaled or nebulized formulations, which could achieve therapeutically relevant local concentrations while minimizing systemic exposure.^67^ This delivery flexibility, combined with the demonstrated synergy with conventional antibiotics, suggests promising potential for clinical applications, particularly in conditions such as CF where localized respiratory tract delivery would be advantageous.

Our current study demonstrates the effectiveness of phenylboronic acid-modified PEI in targeting glycans and preventing bacterial adhesion. However, future modifications could further enhance the selectivity of this approach in the CF environment. With regard to potential strategies for selectively blocking *P. aeruginosa* in cystic fibrosis, it is noteworthy that CF airways often exhibit a slightly acidic microenvironment (pH ∼6.57) due to dysregulated ion transport and persistent infections.^68^ Furthermore, mucins recovered from CF airways are enriched with sialylated glycans, such as sialyl-Lewis x,^69,70^ and blocking sialyl-Lewis x with antibodies has been shown to reduce *P. aeruginosa* binding to airway epithelial cells.^20^ Looking forward, heterocyclic boronic acids could offer an interesting avenue for future research, as they have demonstrated selective binding to sialic acid under mildly acidic conditions.^71^ This property could potentially be leveraged to improve pathogen-specific selectivity, offering a promising alternative approach for blocking *P. aeruginosa* in the CF lung environment.

## Conclusions

In line with recent advancements in developing nanomaterials to combat bacterial infections,^32^ we have successfully developed boronic acid-functionalized polyethyleneimine (PEI-BA) as an effective antibiofilm agent. Through rational polymer design, we transformed a potent but cytotoxic antimicrobial agent (i.e., PEI) into a safe and biocompatible material that effectively prevents bacterial attachment to mucin-rich surfaces. The multivalent presentation of boronic acid groups enables strong, stable interactions with mucin glycans through reversible boronate ester formation, creating a protective barrier that inhibits bacterial colonization. Compared to glycomimetic inhibitors^72^ and antibody-based strategies, our boronic acid-functionalized nanomaterial offers broader applicability and scalability, as it exploits the universal *cis*-diol-binding capability of boronic acids. By targeting mucin rather than specific bacterial lectins, this approach circumvents the challenges of glycomimetic synthesis,^18^ avoids the difficulties associated with anti-glycan antibody production,^73^ and disrupts colonization at its earliest stage. This makes it a more robust and versatile platform for preventing bacterial attachment and biofilm formation.

Our findings reveal that PEI-BA exhibits significant antibiofilm activity through a nonbactericidal mechanism, primarily preventing bacterial adhesion rather than killing bacteria directly. This represents a shift in focus from traditional antimicrobial approaches, potentially reducing the risk of resistance development. Moreover, we demonstrated remarkable synergy between PEI-BA and tobramycin, where the combination treatment significantly enhanced biofilm inhibition compared to either agent alone. This synergistic effect is particularly noteworthy as it addresses two critical clinical challenges: the need for more effective biofilm treatments and the desire to minimize antibiotic exposure to reduce both toxicity risks and resistance development.

Taken together, this study establishes boronic acid-based nanomaterials as promising candidates for biofilm prevention and antibiotic enhancement, paving the way for innovative materials targeting biofilm-associated infections. Looking ahead, this antibiofilm strategy holds promise for several therapeutic applications, particularly in conditions like cystic fibrosis where mucin-bacterial interactions play a critical role. Future research directions could explore optimization of PEI-BA formulations for specific clinical applications, investigation of broader antimicrobial combinations, development of targeted delivery systems, and evaluation of long-term efficacy and safety in more complex biological environments.

## Materials and Methods

### Reagents and Materials

Reagents and solvents were used as obtained without further purification. All solvents were of ACS grade or higher. Supplier product number shown in bold. 3-fluoro-4-formylphenylboronic acid (3-BA) **PC6911** was purchased from Apollo Scientific. Sodium cyanoborohydride (NaBH_3_(CN)) **87839.06** and methanol **M/4056/17** were purchased from Fisher Scientific. 1.8 kDa branched polyethyleneimine (PEI) 50 wt % in H_2_O **408700**, sorbitol **S1876**, fructose **F0127**, PBS tablets **P4417**, type III porcine stomach mucin **M1778**, bovine serum albumin (BSA) **A9418**, crystal violet **C6158**, alizarin red S **A5533**, diethyl ether **24004**, and D_2_O **151882** were purchased from Sigma-Aldrich. Luria–Bertani broth (LB) was composed of 10 g/L tryptone, 5 g/L yeast extract and 10 g/L sodium chloride. *P. aeruginosa* strain used was *P. aeruginosa* ATCC 27853. TLC reaction analysis was performed using precoated silica gel 60 plates from Sigma-Aldrich and visualized using 254 nm UV light and curcumin staining.

### General Synthesis of PEI-BA

PEI-BA was synthesized in PEI monomer:BA ratios of 1:0.4, 1:1, and 1:2, and are referred to as PEI-BA-10%, PEI-BA-25%, and PEI-BA-50% respectively, assuming the predominant position for conjugation is at the primary amine sites of PEI. Below is the synthetic protocol for PEI-BA-25%, however both PEI-BA-10% and PEI-BA-50% were synthesized by adjusting appropriate molar ratios.

### Synthesis of PEI-BA-25%

3-fluoro-4-formylphenylboronic acid (177 mg, 1.05 mmol) was added to methanol (12 mL) and dissolved under sonication and gentle heating (30 °C), followed by addition of sodium cyanoborohydride, NaBH_3_(CN) (94.3 mg, 1.50 mmol). Finally, 1.8 kDa branched polyethyleneimine (PEI) 50 wt % in H_2_O (1 g of supplied solution, 1.06 mmol of branched PEI monomer) was added dropwise and swirled to dissolve. The reaction was stirred under reflux at 40 °C for 92 h. The reaction solution was then concentrated *in vacuo* to ∼2 mL and the crude product precipitated in ice-cold diethyl ether forming an orange gel. Gel precipitates were collected by centrifugation and combined before being dissolved in a minimum volume of Milli Q H_2_O. This solution was added to a dialysis bag (*M*_W_ cutoff 1 kDa) and dialyzed against Milli Q H_2_O for 3 days with the dialysate changed twice daily. The contents of the dialysis bag were then lyophilized to obtain nanomaterial PEI-BA-25%, as an orange solid. Yield = 192.7 mg, 29%.

### Alizarin Red S (ARS) Assays

#### Example Two-Component Assay (ARS and PEI-BA) (Scheme S1)

100 μL of a 200 μM stock of alizarin red S in PBS was added to each well in a row of a 96-well plate. 100 μL of a 1 mM stock of PEI-BA in PBS was added to the final well (column 12) so that this well contained 500 μM PEI-BA and 100 μM ARS. The 1 mM PEI-BA stock was then diluted to 900 μM using PBS, and 100 μL of this was added to the well in column 11. Dilution of the PEI-BA stock continued so that from columns 2–12, measurements of the PEI-BA in 100 μM ARS were performed at 25, 50, 100, 150, 200, 250, 300, 350, 400, 450 and 500 μM. Column 1 contained 0 μM of PEI-BA material. This protocol was performed in triplicate (Figure S4) and with 3-BA in place of PEI-BA (Figure S5).

#### Example Three-Component Assay (ARS, PEI-BA, and Fructose/Sorbitol) (Scheme S2)

100 μL of a 1 mM stock of PEI-BA in 200 μM ARS was added to each well in a row of a 96-well plate. 100 μL of a 90 mM stock of fructose/sorbitol in PBS was added to the final well (column 12) so that this well contained 500 μM PEI-BA, 100 μM ARS and 45 mM fructose/sorbitol. The 90 mM fructose/sorbitol stock was then diluted to 80 mM using PBS, and 100 μL of this was added to the well in column 11. Dilution of the fructose/sorbitol stock continued so that from columns 2–12, measurements of the carbohydrate in 500 μM PEI-BA and 100 μM ARS were performed at 1, 2.5, 5, 10, 15, 20, 30, 35, 40, 45 and 50 mM. Column 1 contained 0 mM of fructose/sorbitol. This protocol was performed in triplicate (Figure S7). A three-component assay was also performed using ARS, 3-BA, and sorbitol (Figure S8). Equations used for calculation BA-glycan binding affinities have been adapted from previous literature^24,44,74^ and can be found in the Supporting Information.

### General Method for Biofilm and Growth Assays

Type III porcine stomach mucin was dissolved in 1X PBS at a final concentration of 1 mg/mL and filter sterilized using a 0.45 μm filter followed by a 0.22 μm filter. To coat the wells of a 96 well plate, 200 μL of the filtered mucin solution was added and the plate allowed to incubate overnight at 4 °C. The next day, the mucin solution was decanted and replaced with 200 μL of sterile 3% BSA and again allowed to incubate overnight at 4 °C. The next day, the 3% BSA was removed, and the wells washed with 200 μL PBS. *P. aeruginosa* and appropriate compounds were added to the wells of the plate at a final OD600 of 0.05 in 1X LB/1X PBS. This was allowed to incubate for 24 h at 37 °C. For biofilm quantification, a crystal violet assay was used in which the *P. aeruginosa* culture was removed and the wells washed with 200 μL 1X PBS twice. To fix the cells, 200 μL of 99% methanol was used and the wells allowed to incubate at room temperature for 15 min. Following incubation, the methanol was decanted and allowed to dry at 37 °C to remove any residual liquid. To stain the wells, 200 μL of 2% crystal violet stain was added and allowed to incubate for 5 min at room temperature. To remove excess crystal violet stain, each well was decanted and rinsed with 200 μL of water six times followed by drying at 37 °C for 10 min. To solubilize the stained wells, 160 μL of 33% acetic acid was added to each well and allowed to incubate for 5 min before measurement of OD570 on a CLARIOStar plate reader.

### Dynamic Light Scattering (DLS) Analyses

#### With Sorbitol

A 1 mg/mL solution of porcine mucin in Milli Q H_2_O was prepared and filtered four times using 0.45 μm syringe filters. A 1 mg/mL solution of mucin in 20 mM sorbitol was prepared in the same way. 1000 μL of the filtered mucin-only solution was added to a 1 × 1 cm^2^ cuvette and particle size measured using DLS. Once measured, to this same cuvette was added 10 μL of a 19 mM PEI-BA-25% solution in H_2_O to make a final PEI-BA-25% concentration of 190 μM. Once added, the cuvette was aspirated and particle size measured after 10 min.

1500 μL of the filtered mucin solution containing 20 mM sorbitol was added to a 1 × 1 cm^2^ cuvette and particle size measured using DLS. Once measured, to this same cuvette was added 15 μL of a 19 mM PEI-BA-25% solution dissolved in 20 mM sorbitol in Milli Q H_2_O. The final concentration in the cuvette of PEI-BA-25% was therefore 190 μM, while the sorbitol concentration remained constant at 20 mM. Once added, the cuvette was aspirated and particle size measured after 10 min.

#### Correlation between Mucin Aggregation and BA Concentration

A 1 mg/mL solution of porcine mucin in Milli Q H_2_O was prepared and filtered four times using 0.45 μm syringe filters. 1000 μL of this filtered solution was added to each of five 1 × 1 cm^2^ cuvettes and particle size of the first cuvette measured using DLS. To the other four were added increasing volumes of a 19 mM PEI-BA-25% solution in H_2_O so that the final concentrations in the cuvettes from cuvette 2 to 5 were 100, 200, 300 and 400 μM of PEI-BA-25% respectively. Once added, the cuvettes were aspirated and particle size measured 10 min after each addition and aspiration (Figure S11).

#### Kinetic Study

A 1 mg/mL solution of porcine mucin in Milli Q H_2_O was prepared and filtered four times using 0.45 μm syringe filters. 1000 μL of this filtered solution was added to a 1 × 1 cm^2^ cuvette and particle size measured using DLS. Once measured, to this same cuvette was added 5 μL of a 19 mM PEI-BA-25% solution in H_2_O to make a final PEI-BA-25% concentration of 95 μM and the solution aspirated. Particle size was then measured after 1 min, and every 5 min after this until 40 min had elapsed. At 35 min, the cuvette was reaspirated prior to measurement (Figure S12).

## Supporting Information Available

Instrumentation used and analytical spectra; figures or schemes not provided in this publication (PDF)
