# Supplementary material for: Phenylboronic Acid-Modified Polyethyleneimine: A Glycan-Targeting Anti-Biofilm Polymer for Inhibiting Bacterial Adhesion to Mucin and Enhancing Antibiotic Efficacy
Source: ACS Appl Mater Interfaces. 2025 Mar 18;17(13):19276–85. doi: 10.1021/acsami.4c20874 (PMC11969427; doi:10.1021/acsami.4c20874)
Supplement: Supplementary file 1 — am4c20874_si_001.pdf [file am4c20874_si_001.pdf]

Supporting Information for

Phenylboronic Acid-Modified Polyethyleneimine: A Glycan-Targeting Anti-Biofilm Polymer for Inhibiting Bacterial Adhesion to Mucin and Enhancing Antibiotic Efficacy

Lorcan J.P. Rooney, [1] *Andrew Marshall,* [2] *Michael M. Tunney,* [2] *Seyed R. Tabaei* [1] *

[1] School of Chemistry and Chemical Engineering, Queen's University Belfast,

David Keir Building, Stranmillis Road, Belfast, BT9 5AG, UK

[2] School of Pharmacy, Queen's University Belfast, Medical Biology Centre,

Lisburn Road, Belfast, BT9 7BL, UK

*Corresponding author s.tabaei@qub.ac.uk

Table of Contents

Instrumentation S2
NMR Spectra of PEI-BA Samples S3
NMR Characterization to Calculate BA-PEI Conjugation S3
PEI-BA-10% S4
PEI-BA-25% S5
PEI-BA-50% S6
Alizarin Red S (ARS) Fluorescence Assays S6
Calculation of Binding Affinities from ARS Assays S6
Two-Component ARS Fluorescence Assays S10
Three-Component ARS Fluorescence Assays S13
DLS Data S18
DLS Concentration of PEI-BA Effect on Mucin Particle Size Study S18
DLS Mucin Particle Size Increase Over Time Study S19
DLS Solubility of PEI-BA-50% Study S20
Anti-Biofilm Assay Control Studies S21
Anti-Biofilm Activity of 3-Fluoro-4-Formylphenylboronic Acid (3-BA) S21
Competitive Binding Biofilm Assay Using Sorbitol S22
References S23

Instrumentation

^{1}H-NMR analyses were performed using a Bruker Ultrashield 400 MHz spectrometer at 25 ˚C. Topspin 4.2.0 NMR software was used for NMR spectra processing. Chemical shifts (δ) are quoted

in parts per million (ppm) against a tetramethylsilane (TMS) standard at 0 ppm in D_2O. Spectra are equilibrated relative to H_2O solvent peaks (^{1}H-NMR 4.79 ppm). ^{1}H-NMR spectra are reported using format δ/ppm (no. of protons, multiplicity, assignment). Fluorescent dye-displacement assays using alizarin red S were performed using an Edinburgh Instruments FS5 spectrofluorometer fitted with an SC-41 plate-reader module, with samples excited at 466 nm and fluorescence recorded at 595 nm. Dynamic light scattering (DLS) analyses were performed using a Malvern Nano-ZS Zen3600 Zetasizer. For biofilm and bacterial growth assays, plates were analysed using a BMG Labtech CLARIOstar plate reader.

NMR Spectra of PEI-BA Samples

NMR Characterization to Calculate BA-PEI Conjugation

Conjugation success was calculated through comparison of peak integrals seen on ^{1}H-NMR spectra. As polymer NMR spectra can be convoluted, integral start and end points were calculated on analysis of coupling in COSY 2D-NMR (data not shown). A monomer of 1.8 kDa branched PEI has a total of 44 protons visible in NMR analysis as amine protons are not visible due to solvent exchange. Calibrating the PEI peaks as 44 hydrogens, then integrating the aromatic region on the ^{1}H-NMR spectrum, gives the number of aromatic protons per PEI monomer. As the only aromatic protons on the PEI monomer would be from 3-BA, an aromatic proton:PEI proton ratio allowed calculation of PEI-BA conjugation success. For example, a ratio of aromatic proton:PEI

proton ratio of 3:44 would indicate a 1:1 3-BA:PEI monomer ratio as seen in the ^{1}H-NMR spectrum for PEI-BA-25% (**Figure S2**).

PEI-BA-10%

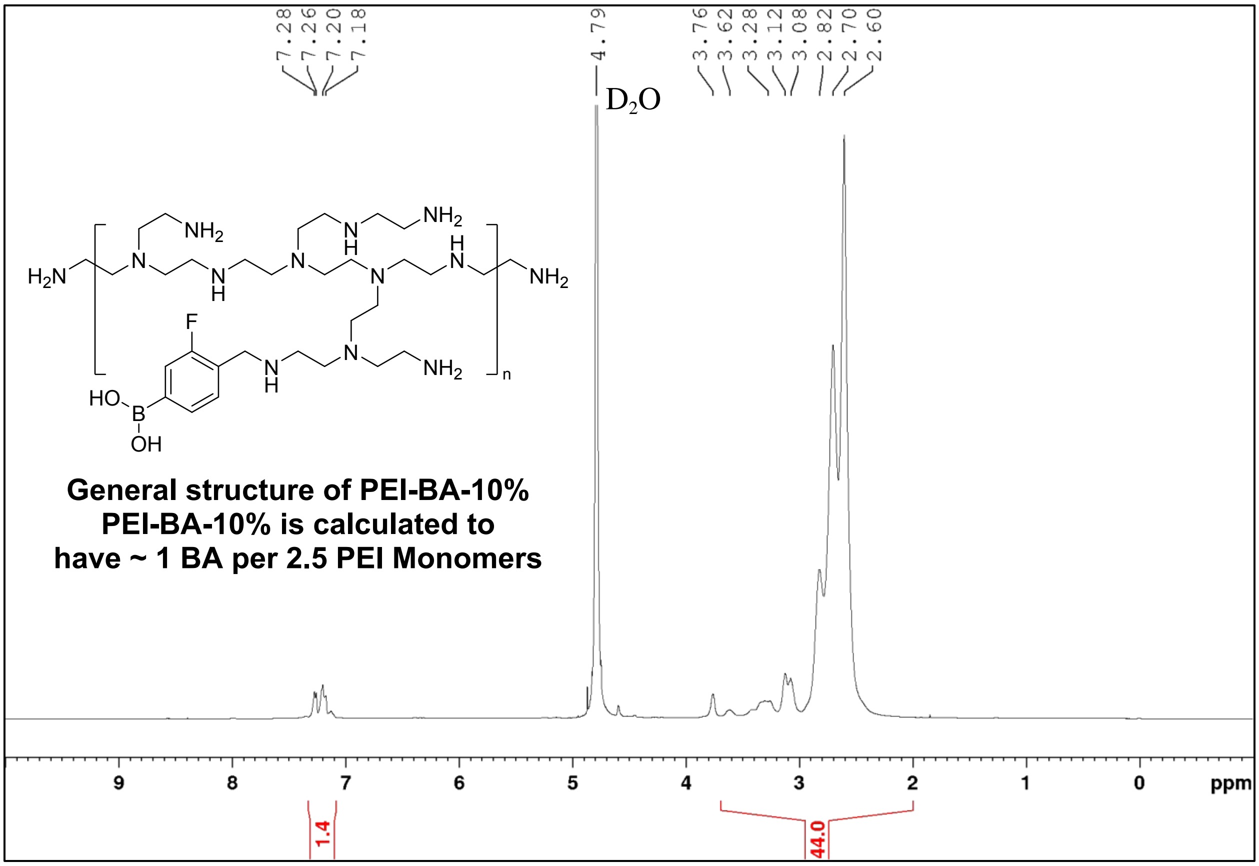

Figure S1. ^{1}H-NMR of PEI-BA-10%. 400 MHz, D_2O: δ = 7.35-7.09 (1.4H, m, 3-BA), 3.70-2.00 (44H, m, PEI monomer).

PEI-BA-25%

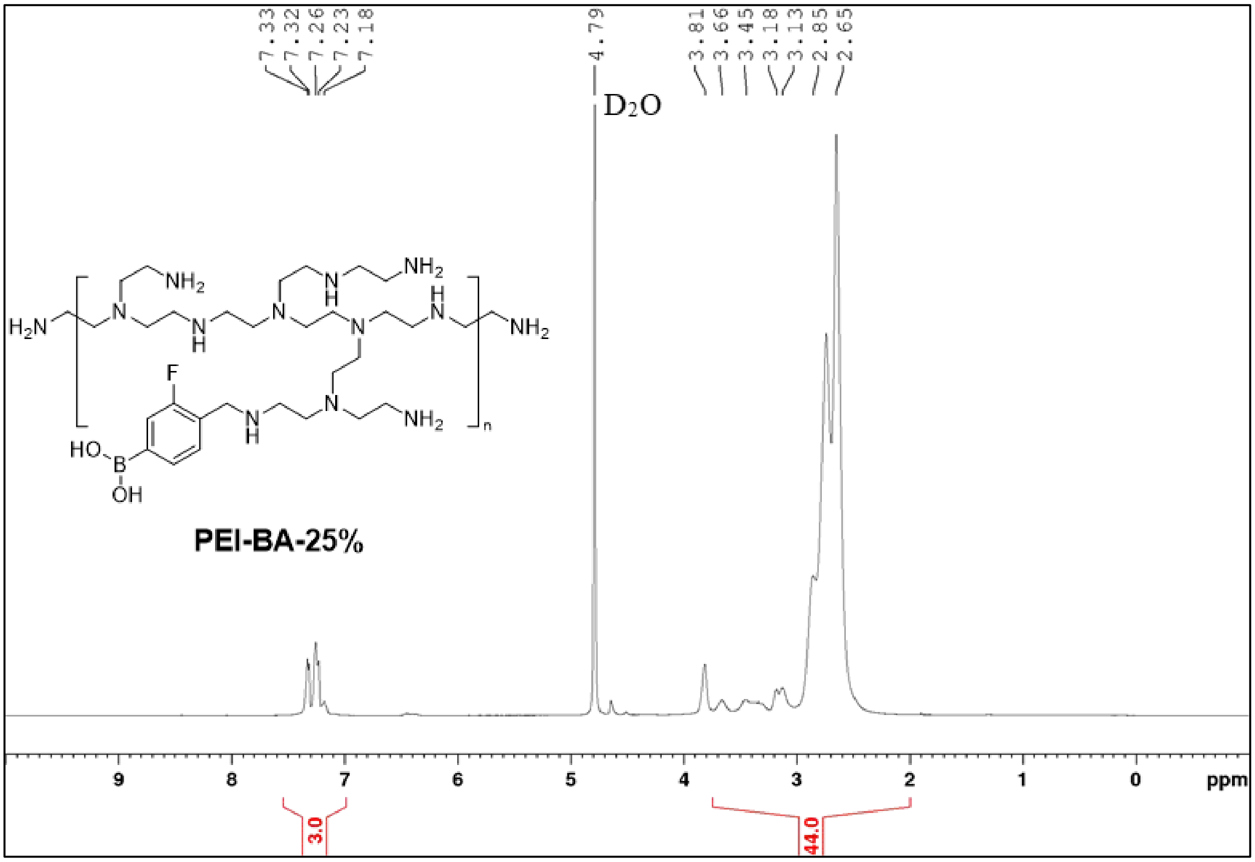

Figure S2. ¹H-NMR of PEI-BA-25%. 400 MHz, D_2O: δ = 7.55-7.00 (3H, m, 3-BA), 3.75-2.00 (44H, m, PEI monomer).

PEI-BA-50%

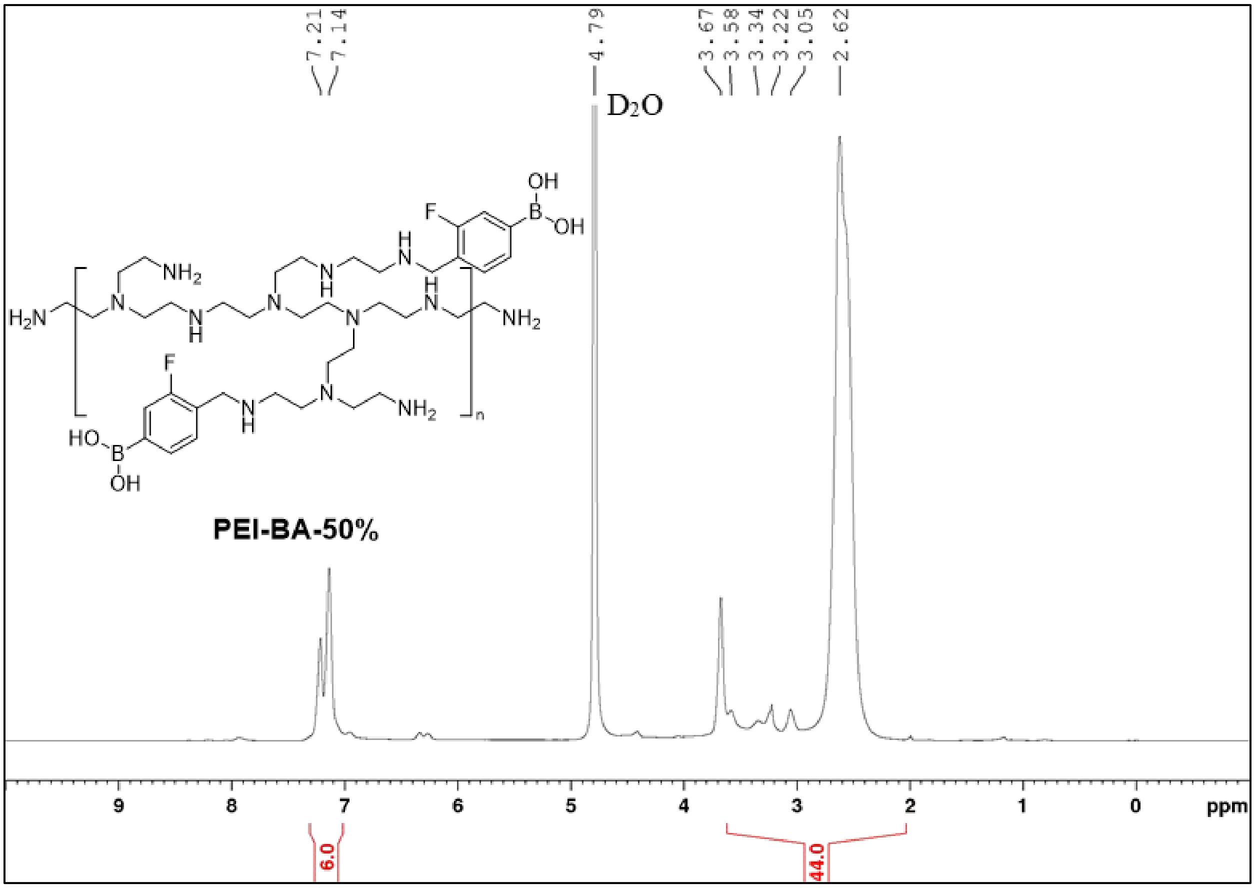

Figure S3. ^{1}H-NMR of PEI-BA-50%. 400 MHz, D_2O: δ = 7.30-7.01 (3H, m, 3-BA), 3.62-2.04 (44H, m, PEI monomer).

Alizarin Red S (ARS) Fluorescence Assays

Calculation of Binding Affinities from ARS Assays

Equations used for calculation binding affinities use the Benesi-Hildebrand method and are adapted from previous literature.[1-3] Any BA samples used are represented in the equations below as “BA”.

The association constant for a two-component system (K_{ARS}) can be written as:

$$(\mathbf{S1})\ \ K_{\mathrm{ARS}} = \frac{[\mathrm{BA{\cdot}ARS}]}{[\mathrm{BA}][\mathrm{ARS}]}$$

Equation (S1) can be rewritten as **equation (S2):**

$$(\mathbf{S2})\ \ K_{\mathrm{ARS}} = \frac{[\mathrm{BA{\cdot}ARS}]}{[\mathrm{BA}]([\mathrm{ARS}]_0 - [\mathrm{BA{\cdot}ARS}])}$$

Rearranging **equation (S2)** gives **equation (S3)**:

$$(\mathbf{S3})\ \ \frac{[\mathrm{ARS}]_0}{[\mathrm{BA{\cdot}ARS}]} = \frac{1}{[\mathrm{BA}]K_{\mathrm{ARS}}} + 1$$

For a given concentration of BA·ARS, the fluorescence intensity (F) is given by **equation (S4)**, where K_{inst} is a constant relating to a specific instrument's parameters such as monochromator throughput, quantum yield, light intensity, molar absorptivity, and path length.

$$(\mathbf{S4})\ \ \mathrm{F} = K_{\mathrm{inst}}[\mathrm{BA{\cdot}ARS}]$$

Rearranging **equation (S4)** gives **equation (S5)** which allows calculation of BA·ARS complex concentration provided all fluorometer instrument parameters are known. Substituting **equation (S5)** into **equation (S3)** gives **equation (S6)**:

$$(\mathbf{S5})\ \ [\mathrm{BA{\cdot}ARS}] = \frac{\mathrm{F}}{K_{\mathrm{inst}}}$$

$$(\mathbf{S6})\ \ \frac{K_{\mathrm{inst}}[\mathrm{ARS}]_0}{\mathrm{F}} = \frac{1}{[\mathrm{BA}]K_{\mathrm{ARS}}} + 1$$

Equation (S6) can be rearranged to give **equation (S7)**, the Benesi-Hildebrand equation:

$$(\mathbf{S7})\ \ \frac{1}{\mathrm{F}} = \frac{1}{K_{\mathrm{inst}}[\mathrm{ARS}]_0 K_{\mathrm{ARS}}[\mathrm{BA}]} + \frac{1}{K_{\mathrm{inst}}[\mathrm{ARS}]_0}$$

If one were to use a constant ARS concentration, then the association constant for a two-component system, K_{ARS} can be calculated from the plot of 1/F vs. 1/[BA]. From this plot, K_{ARS} = intercept/slope. These plots are shown in **Figure S6.**

With K_{ARS} calculated, a three-component system can then be employed to find the association constant of a BA for a sugar (K_{Sug}). In this three-component system, the total concentration of the individual components can be represented by **equations (S8), (S9)** and **(S10)**.

$$(\mathbf{S8})\ \ [\mathrm{sugar}]_0 = [\mathrm{sugar}] + [\mathrm{BA{\cdot}sugar}]$$

$$(\mathbf{S9})\ \ [\mathrm{ARS}]_0 = [\mathrm{ARS}] + [\mathrm{BA{\cdot}ARS}]$$

$$(\mathbf{S10})\ \ [\mathrm{BA}]_0 = [\mathrm{BA}] + [\mathrm{BA{\cdot}sugar}] + [\mathrm{BA{\cdot}ARS}]$$

Like the two-component system and **equation (S1)**, K_{Sug} can be written as **equation (S11)**:

$$(\mathbf{S11})\ \ K_{\mathrm{Sug}} = \frac{[\mathrm{BA{\cdot}sugar}]}{[\mathrm{BA}][\mathrm{sugar}]}$$

Combining **equations (S8-S10)** with **equation (S1)** and **equation (S11)** gives **equation (S12)**:

$$(\mathbf{S12})\ [\mathrm{BA}]_0 = [\mathrm{BA}] + \frac{K_{\mathrm{Sug}}[\mathrm{BA{\cdot}sugar}]}{1 + K_{\mathrm{Sug}}[\mathrm{BA}]} + \frac{K_{\mathrm{ARS}}[\mathrm{BA}][\mathrm{ARS}]_0}{1 + K_{\mathrm{ARS}}[\mathrm{BA}]}$$

If the ARS indicator ratio, Q, is defined using **equation (S13)**, then **equation (S12)** can be rewritten as **equation (S14)**:

$$(\mathbf{S13})\ \ \mathrm{Q} = \frac{[\mathrm{ARS}]}{[\mathrm{BA{\cdot}ARS}]}$$

$$(\mathbf{S14})\ \ [\mathrm{BA}]_0 = \frac{1}{\mathrm{Q}K_{\mathrm{ARS}}} + \frac{K_{\mathrm{Sug}}[\mathrm{sugar}]_0}{\mathrm{Q}K_{\mathrm{ARS}} + K_{\mathrm{Sug}}} + \frac{[\mathrm{ARS}]_0}{1 + \mathrm{Q}}$$

If a value, P, is given by **equation (S15)**, then we can use this to rewrite **equation (S14)** as **equation (S16)**.

$$(\mathbf{S15})\ \ \mathrm{P} = [\mathrm{BA}]_0 - \frac{1}{\mathrm{Q}K_{\mathrm{ARS}}} - \frac{[\mathrm{ARS}]_0}{1 + \mathrm{Q}}$$

$$(\mathbf{S16})\ \ \mathrm{P} = \frac{K_{\mathrm{Sug}}[\mathrm{sugar}]_0}{\mathrm{Q}K_{\mathrm{ARS}} + K_{\mathrm{Sug}}}$$

Equation (S16) can then be rewritten as **equation (S17)**.

$$(\mathbf{S17})\ \ \frac{[\mathrm{sugar}]_0}{\mathrm{P}} = \frac{K_{\mathrm{ARS}}}{K_{\mathrm{Sug}}}\mathrm{Q} + 1$$

The association constant for a three-component system, K_{Sug} can be calculated from the plot of $[\mathrm{sugar}]_o$/P vs. Q. Dividing K_{ARS} by the slope of this plot gives K_{Sug}, and K_d can be calculated by $1/K_{\mathrm{Sug}}$. These plots are shown in **Figure S9** and **Figure S10**.

Q can be obtained from **equation (S18)** using a three-component system where $F_{BA\cdot ARS}$ is the fluorescence intensity of the BA-ARS adduct in the absence of sugar, F_{ARS} is the fluorescence intensity of ARS, and F_{diol} is the measured fluorescence intensity in the presence of the competitive diol.

$$(\mathbf{S18})\quad Q = \frac{(F_{BA\cdot ARS} - F_{diol})}{F_{diol} - F_{ARS}}$$

Two-Component ARS Fluorescence Assays

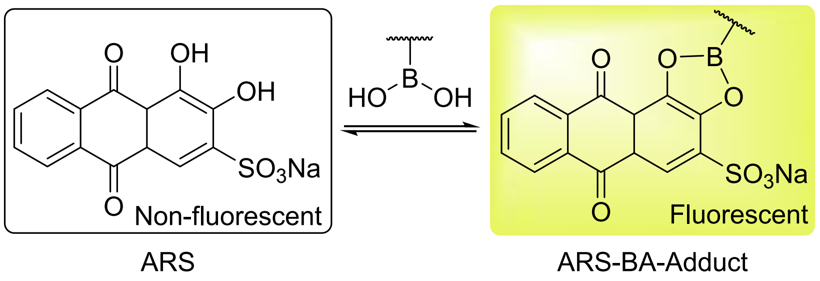

Scheme S1. General scheme for 2-component ARS dye-displacement fluorescence assay using boronic acids.

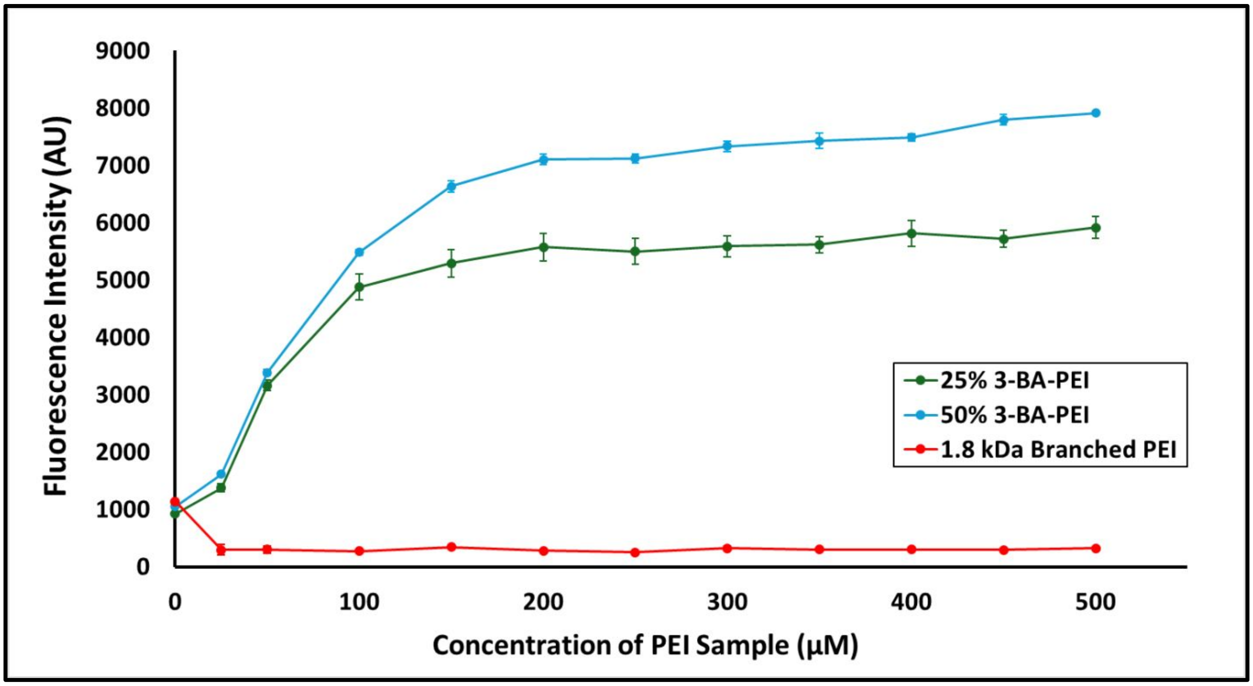

Figure S4. Two-component ARS fluorescence assay with PEI-BA and 1.8 kDa PEI. PEI-BA binds to the *cis*-diol present in ARS' structure via the boronic acid moieties to form a fluorescent PEI-BA-ARS adduct (**Scheme S1**). PEI-BA-50% shows greater fluorescence than PEI-BA-25% at similar sample concentrations, confirming increased BA-functionalization enhances multivalent interactions. Performed in PBS, ARS 100 µM, exc. 466 nm, em. 595 nm, n=3. The high initial fluorescence intensity of ~ 1000 a.u. for 0 µM in all three data series is caused by a difference in the surface tension and menisci of solutions in wells that did not contain PEI or PEI-BA. This affects the fluorescence detection by the spectrofluorometer due to changes in the emission through a curved liquid surface.[4, 5]

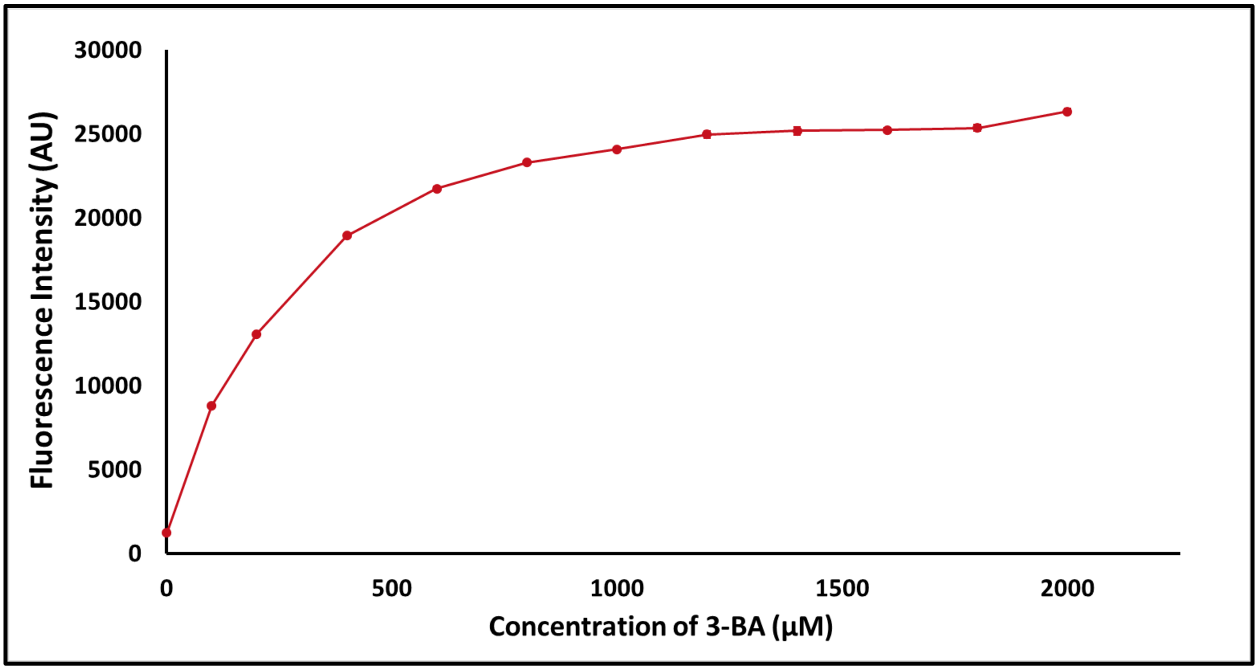

Figure S5. Two-component ARS fluorescence assay with 3-BA. 3-BA binds to the cis-diol present in ARS' structure via the boronic acid moieties to form a fluorescent BA-ARS adduct (**Scheme S1**). Performed in PBS, ARS 100 μM, exc. 466 nm, em. 595 nm, n=3.

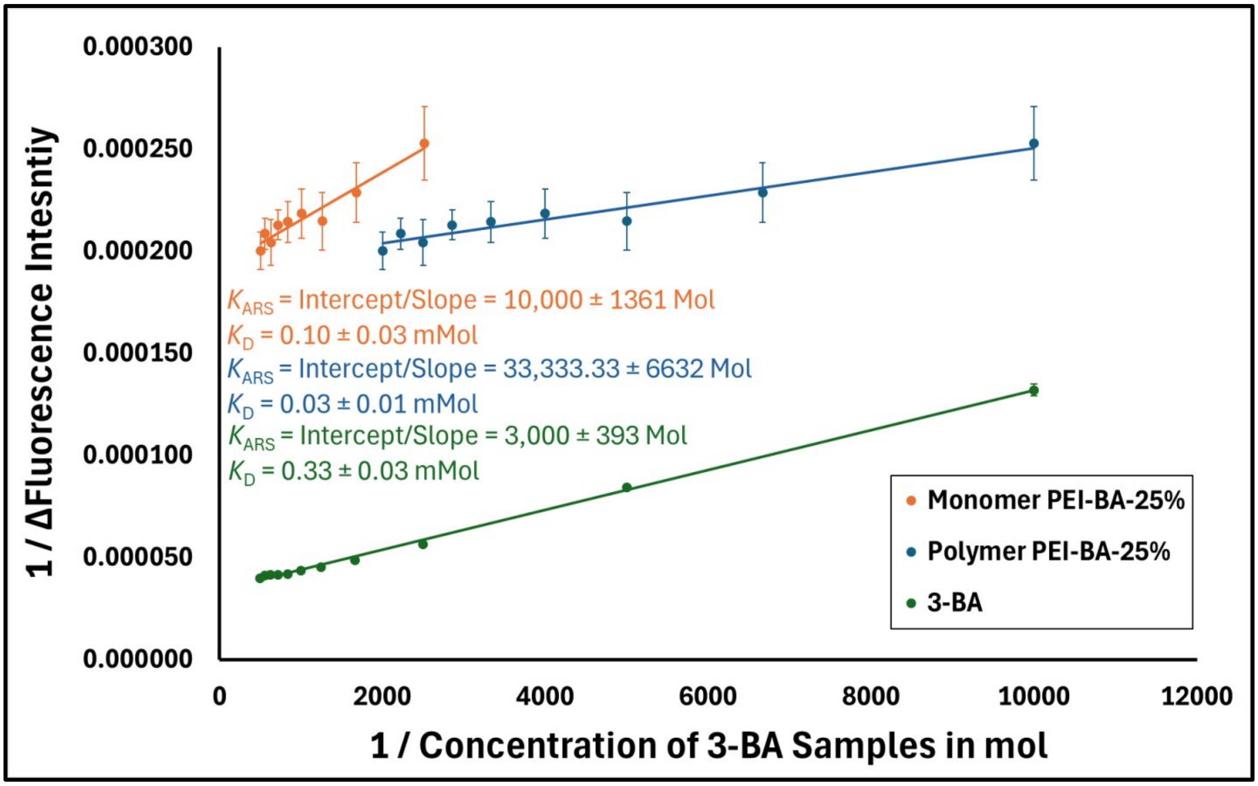

Figure S6. Graph used for the calculation of binding constants between BA-containing samples and ARS. The association constant, K_{ARS}, can be calculated by the formula intercept/slope using the trendlines shown.

Three-Component ARS Fluorescence Assays

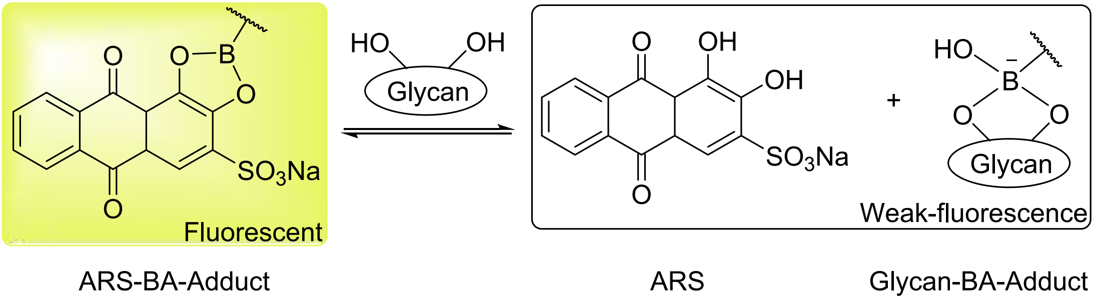

Scheme S2. General scheme for 2-component ARS dye-displacement fluorescence assay using boronic acids.

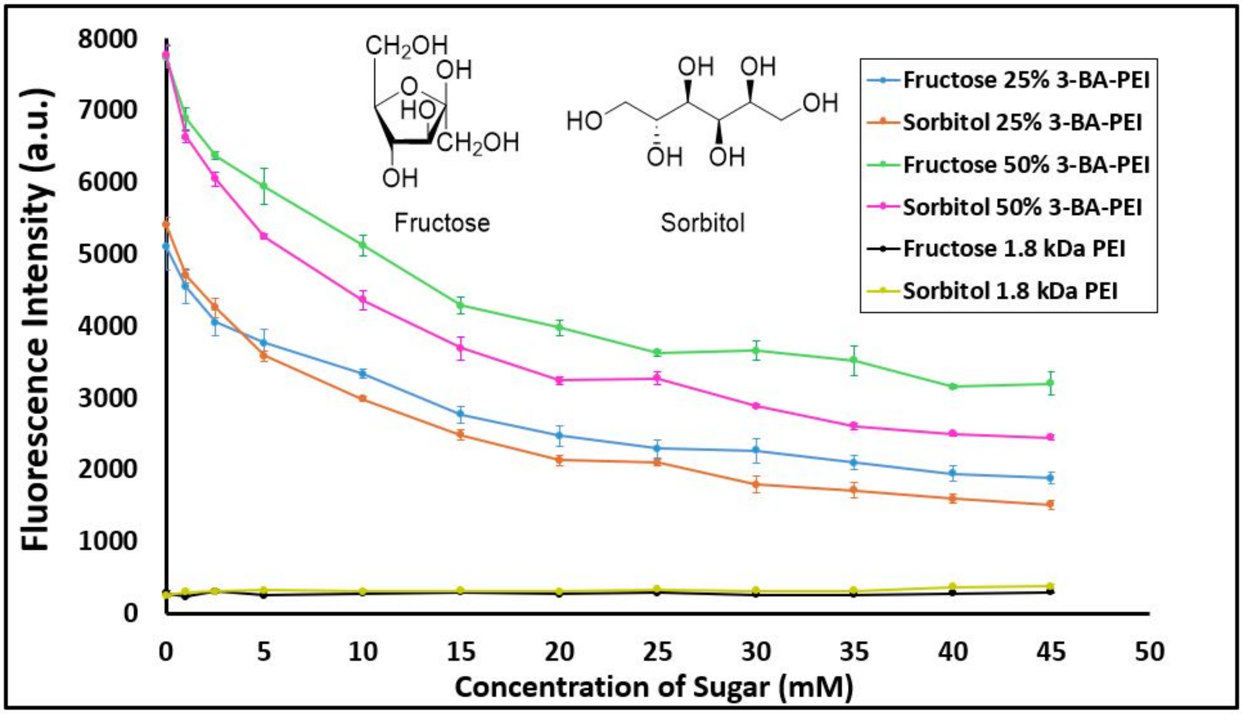

Figure S7. Three-component ARS fluorescence assay. The addition of fructose or sorbitol to a solution of PEI-BA and ARS disrupts the BA-ARS adduct and reduces fluorescence as BA-glycan adducts are formed and non-fluorescent ARS is released (**Scheme S2**). Performed in PBS, sample concentration 500 μM, ARS 100 μM, exc. 466 nm, em. 595 nm, n=3. The addition of sorbitol to PEI-BA-ARS causes a greater fluorescence decrease than fructose as sorbitol has a greater number of *cis*-diols of suitable geometry to allow BA-glycan binding (inset). The addition of fructose or sorbitol to 1.8 kDa PEI alone causes no fluorescence changes as the PEI has no boronic acid moieties present in its structure.

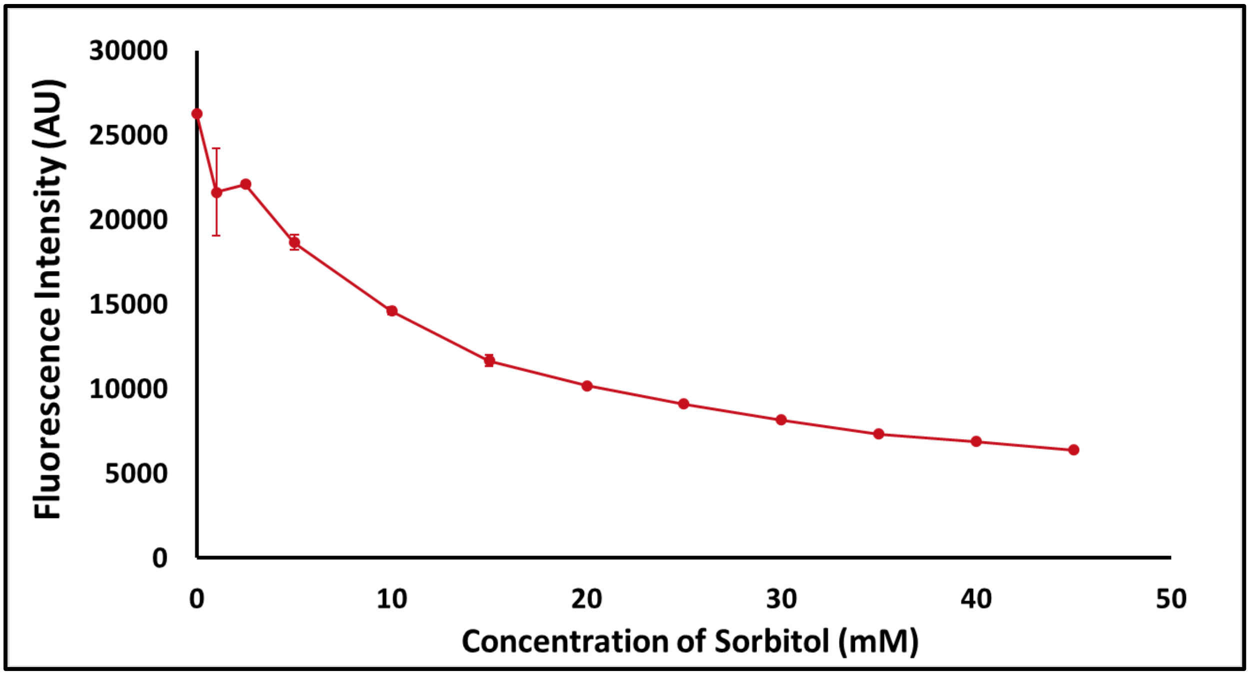

Figure S8. Three-component ARS fluorescence assay with 3-BA and sorbitol. The addition of sorbitol to a solution of 3-BA and ARS disrupts the BA-ARS adduct and reduces fluorescence as BA-sorbtiol adducts are formed and non-fluorescent ARS is released (**Scheme S2**). Performed in PBS, 3-BA 2 mM, ARS 100 μM, exc. 466 nm, em. 595 nm, n=3.

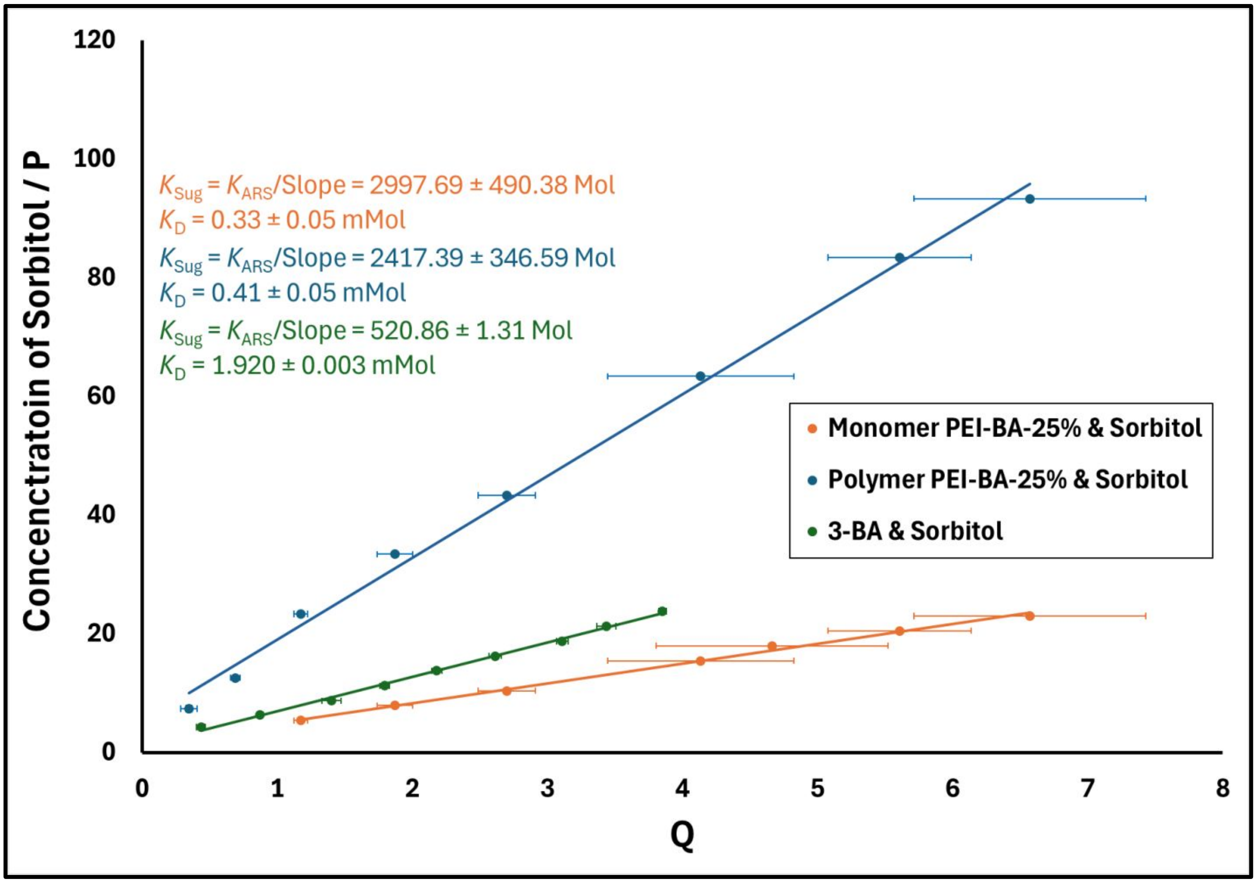

Figure S9. Graph used for the calculation of binding constants between BA-containing samples and sorbitol. The association constant, K_{Sug}, can be calculated by the formula K_{ARS}/Slope using the trendline shown. Values P and Q are derived using **equation (S16)** and **equation (S18)** respectively.

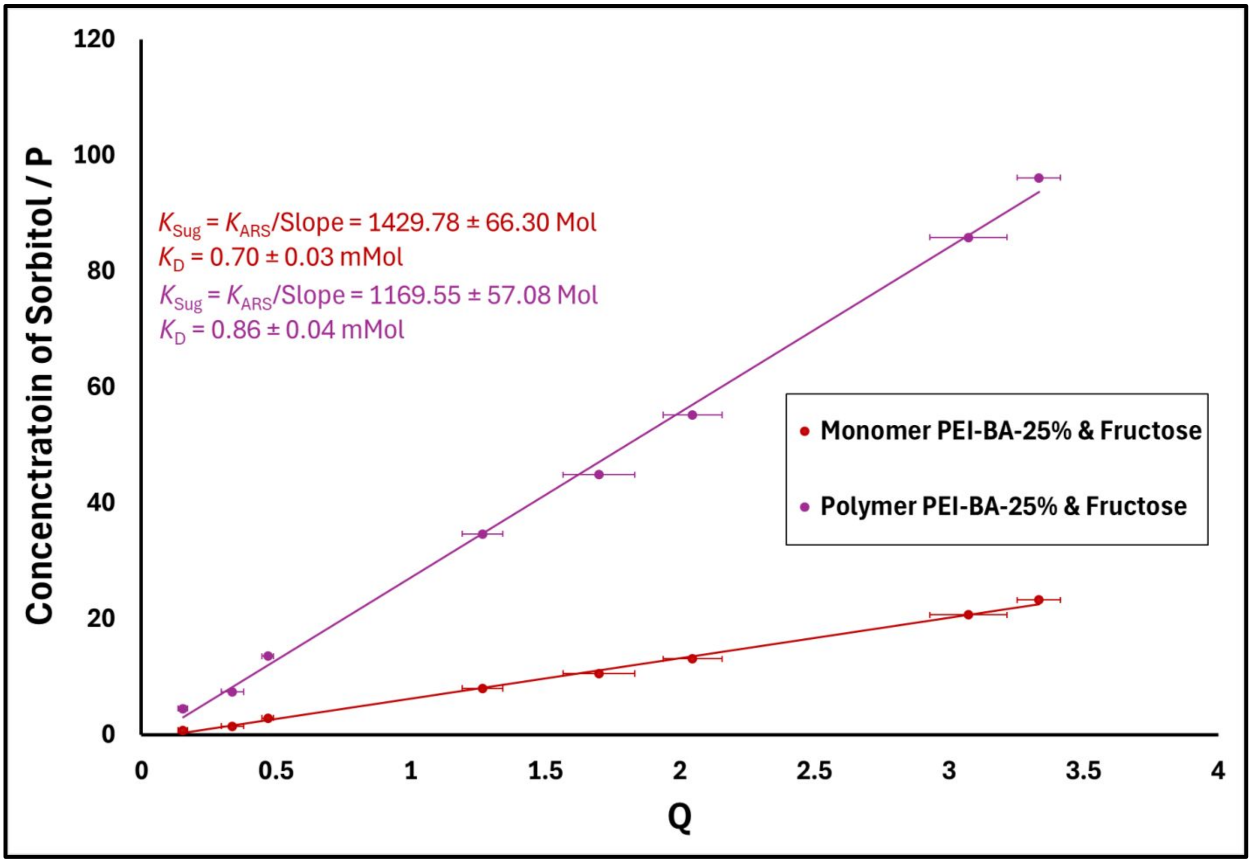

Figure S10. Graph used for the calculation of binding constants between PEI-BA-25% and fructose. The association constant, K_{Sug}, can be calculated by the formula K_{ARS}/Slope using the trendline shown. Values P and Q are derived using **equation (S16)** and **equation (S18)** respectively.

DLS Data

DLS Concentration of PEI-BA Effect on Mucin Particle Size Study

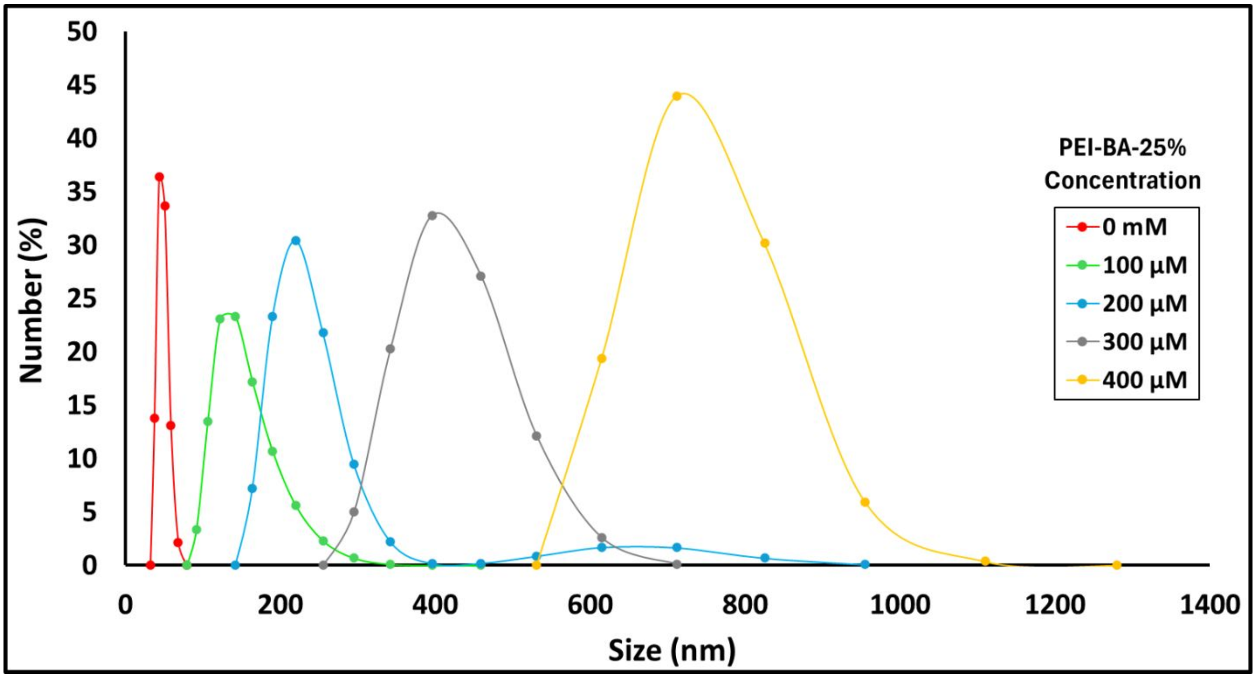

Figure S11. DLS data showing the positive correlation between PEI-BA concentration and mucin aggregation due to increased boronic acid-mucin glycan interactions.

DLS Mucin Particle Size Increase Over Time Study

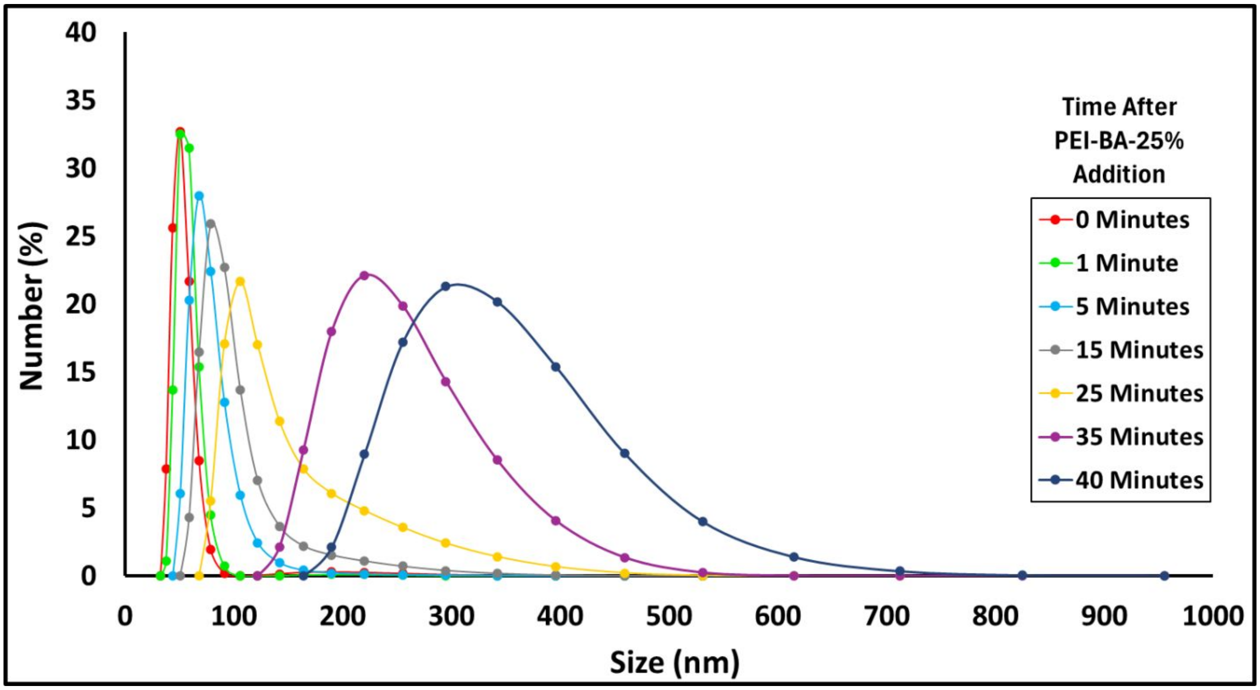

Figure S12. DLS data showing increasing mucin aggregation over time following the addition of PEI-BA-25%. The cuvette was aspirated at 35 minutes to improve suspension homogeneity.

DLS Solubility of PEI-BA-50% Study

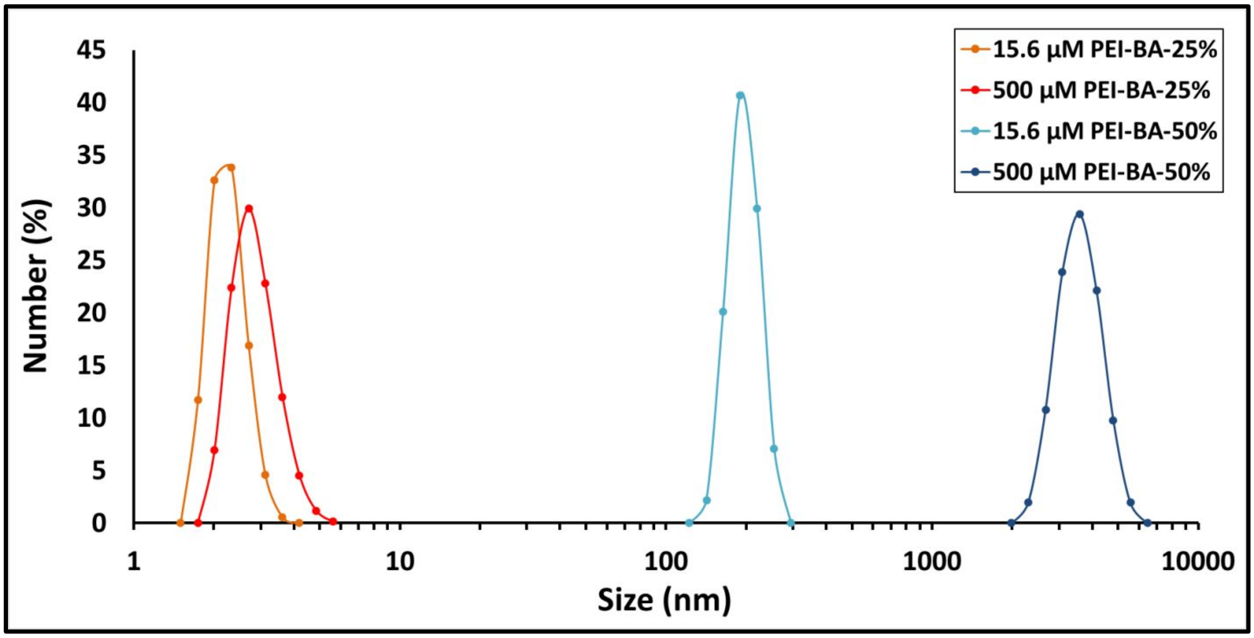

Figure S13. DLS data showing the differences in particle size between similar concentrations of PEI-BA samples. At PEI-BA-25% concentrations of 15.6 µM and 500 µM, particle size was 2-3 nm. At PEI-BA-50% concentrations of 15.6 µM and 500 µM, particle size increased over 18-fold from 190 nm to 3580 nm, respectively. These concentrations represent the lower and upper concentrations of PEI-BA samples tested for anti-biofilm effect.

Anti-Biofilm Assay Control Studies

Anti-Biofilm Activity of 3-Fluoro-4-Formylphenylboronic Acid (3-BA)

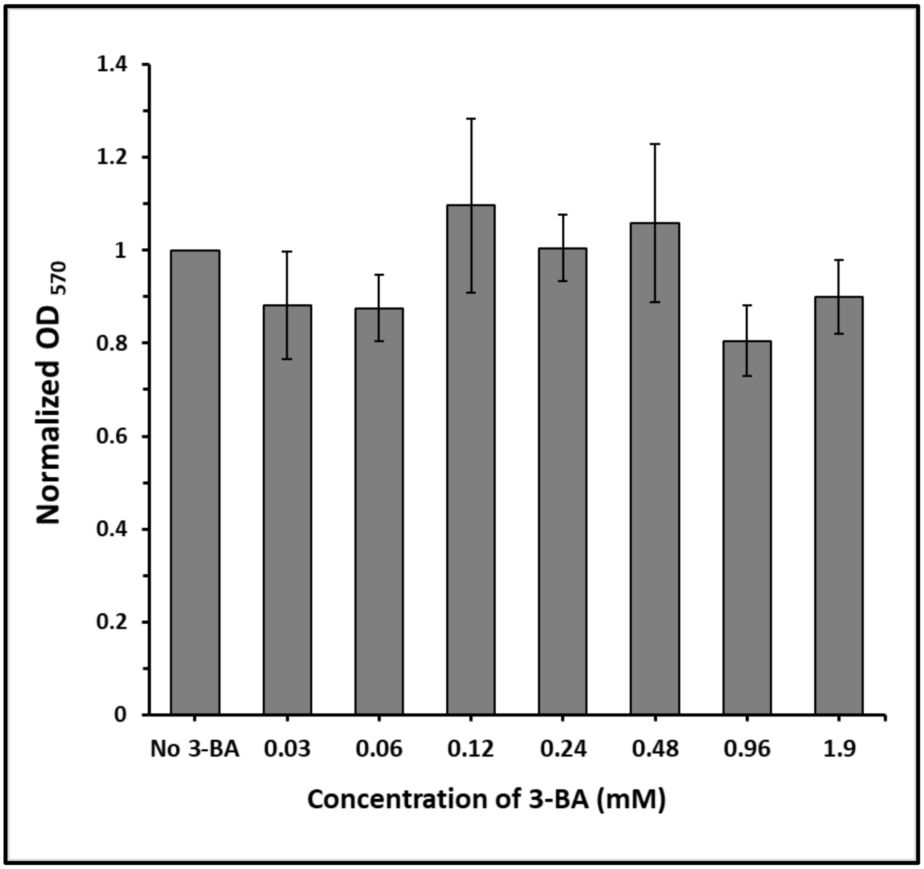

Figure S14. *P. aeruginosa* anti-biofilm activity of 3-BA. The concentrations used are consistent with the concentration of boronic acid moieties present in the PEI-BA-25% tested in Figure 4 of the main text (1:1 ratio of PEI-BA-25% monomer:BA). The data shows that up to 1.9 mM of 3-BA, it is ineffective as an anti-biofilm agent. This demonstrates the anti-biofilm activity of PEI-BA is due to its multivalent scaffold allowing multiple BA-glycan binding events simultaneously.

Competitive Binding Biofilm Assay Using Sorbitol

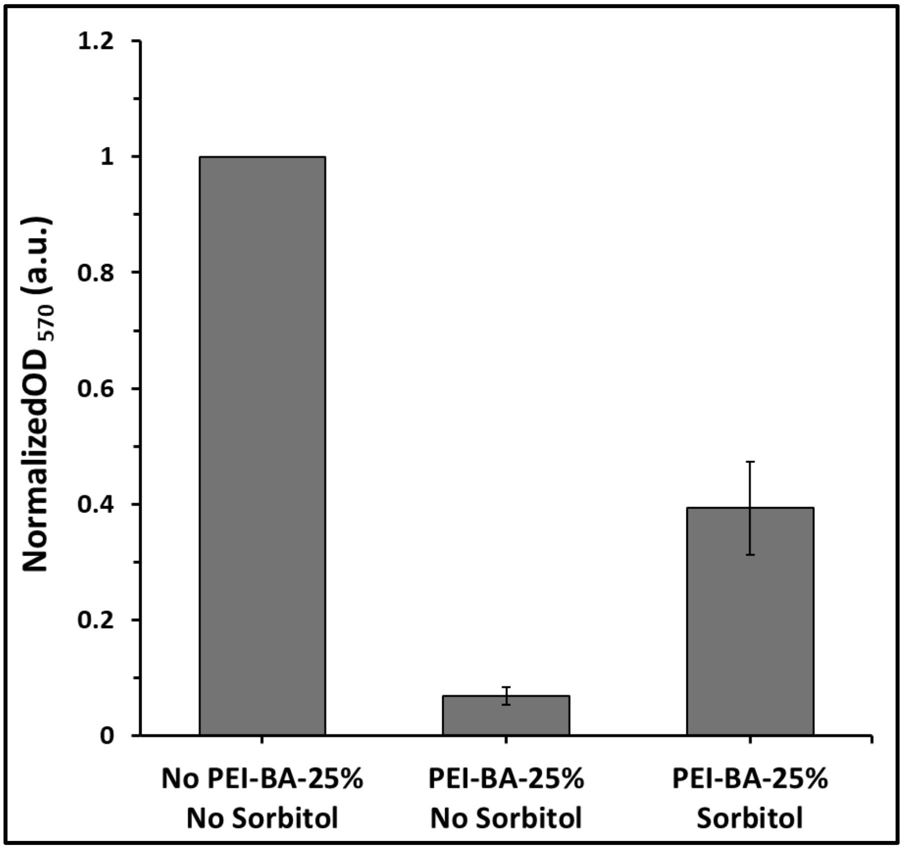

Figure S15. *P. aeruginosa* biofilm formation in the presence of no antibacterial agent (control), in the presence of PEI-BA-25% (250 μM), and in the presence of PEI-BA-25% (250 μM) and sorbitol (20 mM). Sorbitol binds to the BA sites on PEI-BA, preventing the polymer from binding to mucin, which in turn allows increased bacterial growth compared to when no sorbitol is present.

References

(1) Springsteen, G.; Wang, B. A detailed examination of boronic acid–diol complexation. *Tetrahedron* **2002**, *58* (26), 5291-5300. DOI: 10.1016/S0040-4020(02)00489-1.

(2) Benesi, H. A.; Hildebrand, J. H. A Spectrophotometric Investigation of the Interaction of Iodine with Aromatic Hydrocarbons. *Journal of the American Chemical Society* **1949**, *71* (8), 2703-2707. DOI: 10.1021/ja01176a030.

(3) Brooks, W. L. A.; Deng, C. C.; Sumerlin, B. S. Structure–Reactivity Relationships in Boronic Acid–Diol Complexation. *ACS Omega* **2018**, *3* (12), 17863-17870. DOI: 10.1021/acsomega.8b02999.

(4) Lifeng, C.; Gochin, M. Colloidal aggregate detection by rapid fluorescence measurement of liquid surface curvature changes in multiwell plates. *J Biomol Screen* **2007**, *12* (7), 966-971. DOI: 10.1177/1087057107306503.

(5) Cottingham, M. G.; Bain, C. D.; Vaux, D. J. T. Rapid method for measurement of surface tension in multiwell plates. *Laboratory Investigation* **2004**, *84* (4), 523-529. DOI: 10.1038/labinvest.3700054.
